# A Parent–Metabolite Middle-Out PBPK Model for Genistein and Its Glucuronide Metabolite in Rats: Integrating Liver and Enteric Metabolism with Hepatobiliary and Enteroluminal Transport to Assess Glucuronide Recycling

**DOI:** 10.3390/pharmaceutics17070814

**Published:** 2025-06-23

**Authors:** Bhargavi Srija Ramisetty, Rashim Singh, Ming Hu, Michael Zhuo Wang

**Affiliations:** 1 Department of Pharmaceutical Chemistry, The University of Kansas, Lawrence, KS 66047, USA; srija.niper@gmail.com; 2 Department of Pharmacological and Pharmaceutical Sciences, University of Houston College of Pharmacy, Houston, TX 77204, USA; rsingh3@central.uh.edu (R.S.); mhu4@central.uh.edu (M.H.)

**Keywords:** PBPK, gut microbiome, enterohepatic circulation, glucuronide recycling, drug transporters, colon targeting

## Abstract

**Background**: Glucuronide recycling in the gut and liver profoundly affects the systemic and/or local exposure of drugs and their glucuronide metabolites, impacting both clinical efficacy and toxicity. This recycling also alters drug exposure in the colon, making it critical to establish local concentration for drugs targeting colon (e.g., drugs for colon cancer and inflammatory bowel disease). **Methods**: In this study, a parent–metabolite middle-out physiologically based pharmacokinetic (PBPK) model was built for genistein and its glucuronide metabolite to estimate the systemic and local exposure of the glucuronide and its corresponding aglycone in rats by incorporating UDP-glucuronosyltransferase (UGT)-mediated metabolism and transporter-dependent glucuronide disposition in the liver and intestine, as well as gut microbial-mediated deglucuronidation that enables the recycling of the parent compound. **Results**: This parent–metabolite middle-out rat PBPK model utilized in vitro-to-in vivo extrapolated (IVIVE) metabolic and transporter clearance values based on in vitro kinetic parameters from surrogate species, the rat tissue abundance of relevant proteins, and saturable Michaelis–Menten mechanisms. Inter-system extrapolation factors (ISEFs) were used to account for transporter protein abundance differences between in vitro systems and tissues and between rats and surrogate species. Model performance was evaluated at multiple dose levels for genistein and its glucuronide. Model sensitivity analyses demonstrated the impact of key parameters on the plasma concentrations and local exposure of genistein and its glucuronide. Our model was applied to simulate the quantitative impact of glucuronide recycling on the pharmacokinetic profiles in both plasma and colonocytes. **Conclusions**: Our study underlines the importance of glucuronide recycling in determining local drug concentrations in the intestine and provides a preliminary modeling tool to assess the influence of transporter-mediated drug–drug interactions on glucuronide recycling and local drug exposure, which are often misrepresented by systemic plasma concentrations.

## 1. Introduction

Glucuronidation is a major phase II metabolic pathway seen in 40–70% of clinically used drugs such as chloramphenicol, diclofenac, irinotecan (CPT-11)/SN-38, and lorazepam [1,2]. In addition to xenobiotics, endogenous compounds like steroid hormones, bile acids, and neurotransmitters also undergo glucuronidation. The glucuronidation reaction is catalyzed by uridine 5′-diphospho-glucuronosyltransferases (UGTs), where the glucuronide moiety from UDP-glucuronic acid is transferred to form a glucuronide conjugate with an endogenous or xenobiotic molecule. The resulting hydrophilic glucuronide is eliminated mainly into the bile and/or urine. Generally, glucuronide metabolites are pharmacologically inactive, but some glucuronides like morphine-6-glucuronide and buprenorphine-3-glucuronide contribute to pharmacological responses [3,4,5]. One notable characteristic of glucuronide metabolites is the regeneration of their aglycone parent molecules in the distal intestine by gut microbial β-glucuronidase (gmGUS) and their subsequent reabsorption back into the systemic circulation. This allows for the enterohepatic recirculation of parent molecules and their glucuronides, increasing the residence time of these molecules by delaying elimination. In the case of elevated gmGUS activity, regenerated aglycones further increase their colonic and systemic exposure, potentially leading to drug-induced intestinal injury and hormone-related diseases such as breast and prostate cancers [6]. Therefore, it is important to understand how gmGUS activity alters colonic and systemic exposure for compounds undergoing glucuronidation.

Phenolic compounds such as flavonoids are often UGT substrates as they can form mono- and di-*O*-glucuronides owing to their multiple hydroxyls [7]. Due to their hydrophilicity, glucuronides have poor passive membrane permeability and require transporters to cross the membrane. Hence, a physiologically based pharmacokinetic (PBPK) model for such a compound would need to incorporate both UGT-mediated metabolism and transporter-mediated processes [2,8,9]. Genistein (GT), a 4′,5,7-trihydroxy isoflavone, is naturally available in its glycoside form as genistin [10,11]. GT is a lipophilic compound with low aqueous solubility (Table 1) that exhibits non-linear pharmacokinetics at higher doses [12]. GT (pK_a1_ = 7.3) also shows pH-dependent solubility, with an increase in solubility at increasing pH [13,14]. Several PK studies reported low oral bioavailability (~20%) of GT owing to its extensive metabolism [8,15,16,17]. Glucuronidation is the predominant pathway for GT metabolism in rats, forming GT glucuronides (GT-glu), which accounts for 90% of the total genistein plasma concentration (genistein plus all metabolites) [12,15,18,19,20]. As such, GT and GT-glu were selected as model compounds to build a parent–metabolite middle-out PBPK model that integrates hepatic and enteric metabolism (glucuronidation/deglucuronidation), as well as hepatobiliary and enteroluminal transport, to simulate systemic and colonic PK profiles of GT (parent) and GT-glu (glucuronide metabolite) and assess the impact of gmGUS and relevant transporters.

Previously, other groups have built PBPK models for genistein in mice, rats, and humans by considering various mechanisms [21,22,23,24]. Schlosser et al. reported a parent–metabolite PBPK model for GT and GT conjugates in rats where all genistein metabolites were lumped together as GT conjugates [23]. Schlosser’s model considered enterohepatic recirculation via biliary excretion and conversion of GT conjugates to GT in the intestine. However, transporter-mediated processes were not modeled and all metabolic processes were mathematically described using first-order rate constants, which were estimated by the top-down model fitting approach. Zager et al. later reported a PBPK model for GT in rats that focused on biliary excretion and simulating the observed concentration-dependent suppression of biliary excretion [22]. However, Zager’s model had the same limitations as Schlosser’s model and did not consider enterohepatic recirculation as the focus was the biliary excretion shortly (<1 h) after systemic GT administration. In 2017, Boonpawa et al. reported a parent–metabolite PBPK model for GT and GT conjugates in humans that included several mono- and di-conjugated glucuronides and sulfates [21].

NA, not applicable; ^a,b^ values adopted based on structural similarity of ^a^ 3,7,4′ THF-7-O-G and ^b^ Apigenin-7-G.

**Table 1:** Drug-related model parameters for Genistein and its glucuronide obtained from the literature.

Parameter	GT	References	GT-Glu	References
Fraction unbound in plasma (%)	1	[25]	71	[23]
Blood to plasma partition coefficient	0.88	[26]	0.61	[23]
pKa’s	7.3, 10.18 and 11.68	[13]	3.82, 8.15 and 11.24	ADMET predictor
Log P	3.04	[27]	0.34	ADMET predictor
Fraction unbound in liver microsome (%)	71.8	ADMET predictor	100	Assumed
Drug intrinsic solubility (mg/mL)	0.024	[28]	NA	
First order absorption rate constant k_a_ (1/h)	4.02	[29]	NA	
In vitro J_max,Mrp2_ (ng/h/mg) ^a^	NA		111	[30]
K_m,Mrp2_ (ng/mL) ^a^	NA		468	
In vitro J_max,Mrp3_ (ng/h/mg)	NA		486	[31]
K_m,Mrp3_ (ng/mL)	NA		351	
In vitro J_max,Bcrp_ (ng/h/mg)	NA		2756	[24]
K_m,Bcrp_ (ng/mL)	NA		3054	
In vitro J_max,Oatp1b2_ (ng/h/mg) ^b^	NA		3240	[9]
K_m,Oatp1b2_ (ng/mL) ^b^	NA		13,512	

NA, not applicable; ^a,b^ values adopted based on structural similarity of ^a^ 3,7,4′ THF-7-O-G and ^b^ Apigenin-7-G.

Boonpawa’s model employed an in vitro-to-in vivo extrapolation (IVIVE) approach to scale in vitro enzyme kinetics (K_m_ and V_max_) obtained from liver and small intestine S9 fractions to in vivo organ clearance, and the model revealed that genistein-7-*O*-glucuronide (G-7G) was the major circulating metabolite contributing to 40–82% of the total plasma genistein concentrations (genistein plus metabolites). However, this model did not consider enterohepatic recirculation or any transporter-mediated processes and has limited use for evaluating the impact of gmGUS and relevant transporters. Also in 2017, Ge et al. reported a new parent–metabolite PBPK model for GT and GT glucuronides in mice that considered enterohepatic recirculation of GT glucuronides generated in the liver and intestine and Bcrp-mediated efflux of GT glucuronides [24]. Like Schlosser’s mode, Ge’s model assumed perfusion-limited kinetics within the liver and intestine, and described the disposition of GT and GT glucuronides using first-order linear kinetics (rather than saturable kinetics like Michaelis–Menten), which were either estimated by the top-down model fitting approach or predicted using in vitro GT glucuronidation kinetics, Bcrp tissue abundances, and in vitro Bcrp efflux kinetics. In addition, Ge’s model did not consider the complexity of intestinal transit, the location of gmGUS activity, or the site of GT reabsorption. To our knowledge, it is yet to be reported for a parent–metabolite PBPK model that integrates hepatic and enteric metabolism (GT glucuronidation/deglucuronidation), hepatobiliary and enteroluminal transport of GT glucuronides, and the use of saturable Michaelis–Menten kinetics.

**Figure 1:**
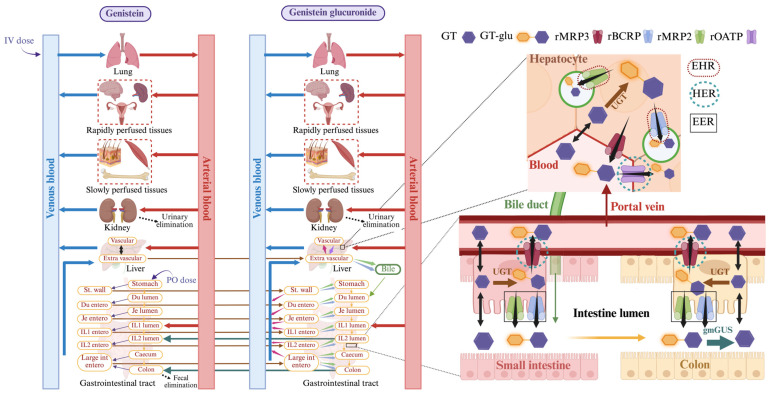
Schematic representation of the parent–metabolite PBPK model for genistein and its glucuronide in rats. Created in BioRender.com.

To better delineate mechanistic processes that enable the overall recycling of GT glucuronides, Hu and coworkers defined three separate recycling pathways occurring in the liver and intestine depending on the location of glucuronide formation [9]. These include enterohepatic recycling (EHR), hepatoenteric recycling (HER), and enteroenteric recycling (EER) (Figure 1). EHR refers to the pathway in which liver-generated glucuronides enter the bile via biliary efflux transporters (e.g., hepatic Mrp2/Bcrp) and convert back to the aglycone in the intestine lumen via gmGUS, and the aglycone reabsorbs back into the systemic circulation before the next round of glucuronidation in the liver. HER refers to the pathway in which intestine-generated glucuronides enter the portal vein via intestinal basolateral efflux transporter (e.g., enteric Mrp3) and the liver via hepatic sinusoidal uptake transporter (e.g., hepatic Oatps; referred to as HER_uptake in our model) and, once in the liver, join the EHR pathway like liver-generated glucuronides. EER refers to the pathway in which intestine-generated glucuronides enter the intestinal lumen via intestinal apical efflux transporters (e.g., enteric Mrp2/Bcrp) and convert back to the aglycone in the intestine lumen via gmGUS, and the aglycone reabsorbs back into enterocytes before the next round of glucuronidation in the intestine. As depicted in Figure 1, in addition to gmGUS activity, transporter-mediated uptake and efflux of GT glucuronides in the liver and intestine are crucial for glucuronide recycling.

Due to the lack of data for in vitro kinetics for relevant rat transporters and transporter protein abundances in in vitro systems used to measure uptake/efflux kinetics from the literature, it is currently untenable to build a fully mechanistic in vitro-to-in vivo extrapolation (IVIVE)-based PBPK model for GT and GT-glu in rats. Instead, the current study aimed to build a parent–metabolite middle-out PBPK model that incorporates relevant mechanistic processes for GT and GT-glu (including hepatic and intestinal glucuronidation of GT, transporter-mediated hepatobiliary and enteroluminal disposition of GT-glu, and gmGUS-dependent conversion of GT-glu back to GT) and to model these mechanistic processes using saturable Michaelis–Menten kinetics, assuming that K_m_ values of rat transporters are similar to those of orthologous transporters from surrogate species and V_max_ values can be scaled from surrogate species to rats by applying an inter-system extrapolation factor (ISEF) for each transporter to account for protein abundance differences between in vitro systems and tissues and inter-species differences in the maximal activity of the relevant transporter. Initial model parameterization was achieved using IVIVE of GT glucuronidation and GT-glu deglucuronidation measured in rat liver and intestinal microsomes and rat feces, rat tissue transporter protein abundances, and kinetic data from surrogate species, followed by fitting the initial model to literature-reported GT/GT-glu plasma concentration–time profiles in rats to determine ISEFs for relevant transporters. Next, the fitted model was evaluated for performance in predicting cumulative urinary excretions following oral administration of GT in rats and predicting GT/GT-glu plasma concentration–time profiles at different doses in rats. Finally, the fitted model was used to assess the influence of gmGUS activity and various transporter-mediated glucuronide recycling pathways on the systemic plasma and local colonocyte exposures of GT and GT-glu in rats to gain insights that can guide future experimental studies and mechanistic PBPK modeling of GT and GT-glu.

## 2. Methods

### 2.1. PBPK Model Building

Our whole-body (WB) PBPK model for genistein (GT) and its glucuronide metabolite (GT-glu) consisted of seven tissues, including lung, liver, kidney, stomach, intestine, rapidly perfused tissues, and slowly perfused tissues (muscle, skin, and fat), as shown in Figure 1. Rapidly perfused tissue volume represents a collective volume of organs with high blood flow such as the heart, brain, spleen, pancreas, and adrenals. The intestine was structured as a six-compartment absorption model based on the advanced compartmental absorption and transit (ACAT) model by incorporating solubility, dissolution, and absorption rate constant parameters adopted from the literature [29,32]. The model can be described by the following differential equations, which were solved by the solver type Ordinary Differential Equations (ODEs) 23t (moderate stiff/trapezoidal rule) [32,33]. For clarification, equations under “Tissue” are for GT and equations under “Tissue_Glu” are for GT-glu.

#### 2.1.1. Liver


(1) $$\begin{array}{c} \frac{{dC}_{liver\;vascular}}{dt}=\frac{1}{V_{liver\;vascular}}(\left(Q_{liver}\left(C_{arterial}-C_{liver\;vascular}\right)\right)+Q_{stomach}\times \frac{\left.{C_{stomach}\times R}_{B:P}\right.}{K_{p,GIT}}+Q_{Intestine}\times \\ \frac{\left.{C_{Intestine}\times R}_{B:P}\right.}{K_{p,GIT}}-{PS}_{Li}\times \left(\frac{\left.C_{liver\;vascular}\times f_{u,p}\right.}{R_{B:P}}-\frac{\left.C_{liver\;extravascular}\times f_{u,p}\right.}{K_{p,liver}}\right)) \end{array}$$

(2) $$\frac{{dC}_{liver\;extravascular}}{dt}=\frac{1}{V_{liver\;extravascular}}\left({PS}_{Li}\times \left(\frac{\left.C_{liver\;vascular}\times f_{u,p}\right.}{R_{B:P}}-\frac{\left.C_{liver\;extravascular}\times f_{u,p}\right.}{K_{p,liver}}\right)-\left(\frac{V_{max,RLM}\times C_{u,liver\;extravascular}}{K_{m,RLM}+C_{u,liver\;extravascular}}\right)\right)$$

#### 2.1.2. Liver_Glu


(3) $$\begin{array}{c} \frac{{dC}_{liver\;vascular\_Glu}}{dt}=\frac{1}{V_{liver\;vascular}}(\left(Q_{liver}\left(C_{arterial\_Glu}-C_{liver\;vascular\_Glu}\right)\right)+Q_{stomach}\times \\ \frac{\left.{C_{stomach\_Glu}\times R}_{B:P\_Glu}\right.}{\left(K_{p,GIT\_Glu}\times K_{p,scalar\_Glu}\right)}+Q_{Intestine}\times \frac{\left.{C_{Intestine\_Glu}\times R}_{B:P\_Glu}\right.}{\left(K_{p,GIT\_Glu}\times K_{p,scalar\_Glu}\right)}-\\ \left(\frac{\left(J_{max,Oatp1b2}\times {Oatps}_{i}\times {ISEF}_{uptake}\right)\times C_{u,liver\;vascular\_Glu}}{K_{m,Oatp1b2}+C_{u,liver\;vascular\_Glu}}\right)+\left(\frac{\left(J_{max,Mrp3}\times {Mrp3}_{i}\times {ISEF}_{Mrp3}\right)\times C_{u,liver\;extravascular\_Glu}}{K_{m,Mrp3}+C_{u,liver\;extravascular\_Glu}}\right)) \end{array}$$

(4) $$\begin{array}{c} \frac{{dC}_{liver\;extravascular\_Glu}}{dt}=\frac{1}{V_{liver\;extravascular}}(\left(\frac{V_{max,RLM}\times C_{u,liver\;extravascular}}{K_{m,RLM}+C_{u,liver\;extravascular}}\right)+\\ \left(\frac{\left(J_{max,Oatp1b2}\times {Oatps}_{i}\times {ISEF}_{uptake}\right)\times C_{u,liver\;vascular\_Glu}}{K_{m,Oatp1b2}+C_{u,liver\;vascular\_Glu}}\right)-\left(\frac{\left(J_{max,Mrp3}\times {Mrp3}_{i}\times {ISEF}_{Mrp3}\right)\times C_{u,liver\;extravascular\_Glu}}{K_{m,Mrp3}+C_{u,liver\;extravascular\_Glu}}\right)-\\ \left(\frac{\left(J_{max,Mrp2}\times {Mrp2}_{i}\times {ISEF}_{Mrp2}\right)\times C_{u,liver\;extravascular\_Glu}}{K_{m,Mrp2}+C_{u,liver\;extravascular\_Glu}}\right)-\left(\frac{\left(J_{max,Bcrp}\times {Bcrp}_{i}\times {ISEF}_{Bcrp}\right)\times C_{u,liver\;extravascular\_Glu}}{K_{m,Bcrp}+C_{u,liver\;extravascular\_Glu}}\right)) \end{array}$$

#### 2.1.3. Bile_Glu


(5) $$\frac{{\mathrm{dC}}_{\mathrm{Bile}\_\mathrm{Glu}}}{\mathrm{dt}}=\frac{1}{V_{\mathrm{Bile}}}\left(\left(\frac{\left(J_{\max,\mathrm{Mrp}2}\times {\mathrm{Mrp}2}_{i}\times {\mathrm{ISEF}}_{\mathrm{Mrp}2}\right)\times C_{u,\mathrm{liver}\;\mathrm{extravascular}\_\mathrm{Glu}}}{K_{m,\mathrm{Mrp}2}+C_{u,\mathrm{liver}\;\mathrm{extravascular}\_\mathrm{Glu}}}\right)+\left(\frac{\left(J_{\max,\mathrm{Bcrp}}\times {\mathrm{Bcrp}}_{i}\times {\mathrm{ISEF}}_{\mathrm{Bcrp}}\right)\times C_{u,\mathrm{liver}\;\mathrm{extravascular}\_\mathrm{Glu}}}{K_{m,\mathrm{Bcrp}}+C_{u,\mathrm{liver}\;\mathrm{extravascular}\_\mathrm{Glu}}}\right)-Q_{\mathrm{bile}}\times \;C_{\mathrm{bile}\_\mathrm{Glu}}\right)$$

#### 2.1.4. Stomach


(6) $$\frac{{\mathrm{dA}}_{\mathrm{undissolved}(\mathrm{ST})}}{\mathrm{dt}}=-\left(\left(\frac{1}{\mathrm{GER}}\right)\;\times A_{\mathrm{undissolved}(\mathrm{ST})}\right)-\left(\mathrm{dissolution}\;\times \;A_{\mathrm{undissolved}(\mathrm{ST})}\;\times \;\left({\mathrm{Solubility}}_{\mathrm{ST}}-C_{\mathrm{dissolved}(\mathrm{ST})}\right)\right)$$

(7) $$\frac{{\mathrm{dA}}_{\mathrm{dissolved}(\mathrm{ST})}}{\mathrm{dt}}=-\left(\left(\frac{1}{\mathrm{GER}}\right)\;\times A_{\mathrm{dissolved}(\mathrm{ST})}\right)+\left(\mathrm{dissolution}\;\times \;A_{\mathrm{undissolved}(\mathrm{ST})}\;\times \;\left({\mathrm{Solubility}}_{\mathrm{ST}}-\;C_{\mathrm{dissolved}(\mathrm{ST})}\right)\right)-k_{a}\times \;A_{\mathrm{dissolved}(\mathrm{ST})}$$

(8) $$\frac{{dC}_{stomach}}{dt}=\frac{1}{V_{stomach}}\left(Q_{stomach}\times \left(C_{arterial}-\frac{\left.C_{stomach}\times R_{B:P}\right.}{K_{p,intestine}}\right)+k_{a,sys}\times A_{u,stomachwall}\right)$$

#### 2.1.5. Duodenum


(9) $$\begin{array}{c} \frac{{\mathrm{dA}}_{\mathrm{undissolved}(\mathrm{Du})}}{\mathrm{dt}}=-\left(\left(\frac{1}{{\mathrm{tranist}}_{\mathrm{Du}}}\right)\;\times A_{\mathrm{undissolved}(\mathrm{Du})}\right)+\left(\left(\frac{1}{\mathrm{GER}}\right)\;\times A_{\mathrm{undissolved}(\mathrm{ST})}\right)-(\mathrm{dissolution}\;\times \;A_{\mathrm{undissolved}(\mathrm{Du})}\;\times \\ \left({\mathrm{Solubility}}_{\mathrm{Int}}-C_{\mathrm{dissolved}(\mathrm{Du})}\right)) \end{array}$$

(10) $$\begin{array}{c} \frac{{\mathrm{dA}}_{\mathrm{dissolved}(\mathrm{Du})}}{\mathrm{dt}}=-\left(\left(\frac{1}{{tranist}_{Du}}\right)\times A_{\mathrm{dissolved}(\mathrm{Du})}\right)+\left(\left(\frac{1}{GER}\right)\times A_{\mathrm{dissolved}(\mathrm{ST})}\right)+(dissolution\times A_{\mathrm{undissolved}(\mathrm{Du})}\times \\ \left({Solubility}_{\mathrm{Int}}-C_{\mathrm{dissolved}(\mathrm{Du})}\right))-k_{a}\times A_{\mathrm{dissolved}(\mathrm{Du})} \end{array}$$

(11) $$\frac{{\mathrm{dC}}_{u,\mathrm{enterocyte}(\mathrm{Du})}}{\mathrm{dt}}=\frac{1}{V_{\mathrm{enterocyte}(\mathrm{Du})}}\left(k_{a}\times A_{\mathrm{dissolved}(\mathrm{Du})}-\left(\frac{V_{max,RDM}\times C_{u,\mathrm{enterocyte}(\mathrm{Du})}}{K_{m,RDM}+C_{u,\mathrm{enterocyte}(\mathrm{Du})}}\right)\right)-k_{a,sys}\times A_{u,\mathrm{enterocyte}(\mathrm{Du})}$$

#### 2.1.6. Jejunum, Ileum, Caecum, and Colon


(12) $$\begin{array}{c} \frac{{\mathrm{dA}}_{\mathrm{undissolved}(n)}}{\mathrm{dt}}=\left(\left(\frac{1}{{tranist}_{n-1}}\right)\times A_{\mathrm{undissolved}(n-1)}\right)-\left(\left(\frac{1}{{tranist}_{n}}\right)\times A_{\mathrm{undissolved}(n)}\right)-(dissolution\times A_{\mathrm{undissolved}(n)}\times \\ \left({Solubility}_{n}-C_{\mathrm{dissolved}(n)}\right)) \end{array}$$

(13) $$\begin{array}{c} \frac{{\mathrm{dA}}_{\mathrm{dissolved}(n)}}{\mathrm{dt}}=\left(\left(\frac{1}{{tranist}_{n-1}}\right)\times A_{\mathrm{dissolved}(n-1)}\right)-\left(\left(\frac{1}{{tranist}_{n}}\right)\times A_{\mathrm{dissolved}(n)}\right)+(dissolution\times A_{\mathrm{undissolved}(n)}\times \\ \left({Solubility}_{n}-C_{\mathrm{dissolved}(n)}\right))-k_{a}\times A_{\mathrm{dissolved}(n)} \end{array}$$

#### 2.1.7. Jejunum, Ileum, and Large Intestine Enterocytes


(14) $$\frac{{\mathrm{dC}}_{u,\mathrm{enterocyte}(n)}}{\mathrm{dt}}=\frac{1}{V_{\mathrm{enterocyte}(n)}}\left(k_{a}\times A_{\mathrm{dissolved}(n)}-\left(\frac{V_{max,RnM}\times C_{u,\mathrm{enterocyte}(n)}}{K_{m,RnM}+C_{u,\mathrm{enterocyte}(n)}}\right)\right)-k_{a,sys}\times A_{u,\mathrm{enterocyte}(n)}$$

#### 2.1.8. Stomach_Glu


(15) $$\frac{{\mathrm{dC}}_{\mathrm{stomach}\_\mathrm{Glu}}}{\mathrm{dt}}=\frac{1}{V_{stomach}}\left(Q_{\mathrm{stomach}}\times \left(C_{\mathrm{arterial}\_\mathrm{Glu}}-\frac{\left.C_{stomach\_Glu}\times R_{B:P\_Glu}\right.}{\left(K_{p,\mathrm{intestine}\_\mathrm{Glu}}\times K_{p,scalar\_Glu}\right)}\right)\right)$$

#### 2.1.9. Duodenum_Glu


(16) $$\begin{array}{c} \frac{{\mathrm{dC}}_{u,\mathrm{enterocyte}(\mathrm{Du})\_\mathrm{Glu}}}{\mathrm{dt}}=\frac{1}{V_{\mathrm{enterocyte}(\mathrm{Du})}}(\left(\frac{V_{max,RDM}\times C_{u,\mathrm{enterocyte}(\mathrm{Du})}}{K_{m,RDM}+C_{u,\mathrm{enterocyte}(\mathrm{Du})}}\right)-\left(\frac{\left(J_{max,Mrp3}\times {Mrp3}_{i}\times {ISEF}_{Mrp3}\right)\times C_{u,\mathrm{enterocyte}(\mathrm{Du})\_\mathrm{Glu}}}{K_{m,Mrp3}+C_{u,\mathrm{enterocyte}(\mathrm{Du})\_\mathrm{Glu}}}\right)-\\ \left(\frac{\left(J_{max,Mrp2}\times {Mrp2}_{i}\times {ISEF}_{apical}\right)\times C_{u,\mathrm{enterocyte}(\mathrm{Du})\_\mathrm{Glu}}}{K_{m,Mrp2}+C_{u,\mathrm{enterocyte}(\mathrm{Du})\_\mathrm{Glu}}}\right)-\left(\frac{\left(J_{max,Bcrp}\times {Bcrp}_{i}\times {ISEF}_{apical}\right)\times C_{u,\mathrm{enterocyte}(\mathrm{Du})\_\mathrm{Glu}}}{K_{m,Bcrp}+C_{u,\mathrm{enterocyte}(\mathrm{Du})\_\mathrm{Glu}}}\right)) \end{array}$$

(17) $$\begin{array}{c} \frac{{\mathrm{dC}}_{\mathrm{lumen}(\mathrm{Du})\_\mathrm{Glu}}}{\mathrm{dt}}=\frac{1}{V_{\mathrm{lumen}(\mathrm{Du})}}(\left(\frac{1}{GER}\right)\times A_{\mathrm{ST}\_\mathrm{Glu}}+\left(\frac{\left(J_{max,Mrp2}\times {Mrp2}_{i}\times {ISEF}_{apical}\right)\times C_{u,\mathrm{enterocyte}(\mathrm{Du})\_\mathrm{Glu}}}{K_{m,Mrp2}+C_{u,\mathrm{enterocyte}(\mathrm{Du})\_\mathrm{Glu}}}\right)+\\ \left(\frac{\left(J_{max,Bcrp}\times {Bcrp}_{i}\times {ISEF}_{apical}\right)\times C_{u,\mathrm{enterocyte}(\mathrm{Du})\_\mathrm{Glu}}}{K_{m,Bcrp}+C_{u,\mathrm{enterocyte}(\mathrm{Du})\_\mathrm{Glu}}}\right)+Q_{bile}\times C_{bile\_Glu}-\left(\frac{1}{{tranist}_{Du}}\right)\times A_{\mathrm{Du}\_\mathrm{Glu}}) \end{array}$$

#### 2.1.10. Lumen and Enterocytes of Jejunum_Glu, Ileum_Glu, and Large Intestine_Glu


(18) $$\begin{array}{c} \frac{{\mathrm{dC}}_{u,\mathrm{enterocyte}(n)\_\mathrm{Glu}}}{\mathrm{dt}}=\frac{1}{V_{\mathrm{enterocyte}(n)}}(\left(\frac{V_{max,RnM}\times C_{u,\mathrm{enterocyte}(n)}}{K_{m,RnM}+C_{u,\mathrm{enterocyte}(n)}}\right)-\left(\frac{\left(J_{max,Mrp3}\times Mrp3\times {ISEF}_{Mrp3}\right)\times C_{u,\mathrm{enterocyte}(n)\_\mathrm{Glu}}}{K_{m,Mrp3}+C_{u,\mathrm{enterocyte}(n)\_\mathrm{Glu}}}\right)-\\ \left(\frac{\left(J_{max,Mrp2}\times Mrp2\times {ISEF}_{apical}\right)\times C_{u,\mathrm{enterocyte}(n)\_\mathrm{Glu}}}{K_{m,Mrp2}+C_{u,\mathrm{enterocyte}(n)\_\mathrm{Glu}}}\right)-\left(\frac{\left(J_{max,Bcrp}\times Bcrp\times {ISEF}_{apical}\right)\times C_{u,\mathrm{enterocyte}(n)\_\mathrm{Glu}}}{K_{m,Bcrp}+C_{u,\mathrm{enterocyte}(n)\_\mathrm{Glu}}}\right)) \end{array}$$

(19) $$\begin{array}{c} \frac{{\mathrm{dC}}_{\mathrm{lumen}(n)\_\mathrm{Glu}}}{\mathrm{dt}}=\frac{1}{V_{\mathrm{lumen}(n)}}(\left(\frac{1}{{tranist}_{n-1}}\right)\times A_{n-1\_\mathrm{Glu}}-\left(\frac{1}{{tranist}_{n}}\right)\times A_{n\_\mathrm{Glu}}+\\ \left(\frac{\left(J_{max,Mrp2}\times {Mrp2}_{i}\times {ISEF}_{apical}\right)\times C_{u,\mathrm{enterocyte}(\mathrm{Du})\_\mathrm{Glu}}}{K_{m,Mrp2}+C_{u,\mathrm{enterocyte}(\mathrm{Du})\_\mathrm{Glu}}}\right)+\left(\frac{\left(J_{max,Bcrp}\times {Bcrp}_{i}\times {ISEF}_{apical}\right)\times C_{u,\mathrm{enterocyte}(\mathrm{Du})\_\mathrm{Glu}}}{K_{m,Bcrp}+C_{u,\mathrm{enterocyte}(\mathrm{Du})\_\mathrm{Glu}}}\right)) \end{array}$$

#### 2.1.11. gmGUS Mediated Regeneration of Genistein in Distal Ileum and Colon


(20) $$\begin{array}{c} \frac{{\mathrm{dA}}_{\mathrm{undissolved}(\mathrm{IL}2)}}{\mathrm{dt}}=\left(\left(\frac{1}{{tranist}_{\mathrm{IL}1}}\right)\times A_{\mathrm{undissolved}(\mathrm{IL}1)}\right)-\left(\left(\frac{1}{{tranist}_{\mathrm{IL}2}}\right)\times A_{\mathrm{undissolved}(\mathrm{IL}2)}\right)-(dissolution\;\times \\ A_{\mathrm{undissolved}(\mathrm{IL}2)}\times \left({Solubility}_{\left(\mathrm{IL}2\right)}-C_{\mathrm{dissolved}(\mathrm{IL}2)}\right))+\left(\frac{V_{max,\beta -glucuronidase}\times C_{u,\mathrm{enterocyte}(\mathrm{IL}2)\_\mathrm{Glu}}}{K_{m,\beta -glucuronidase}+C_{u,\mathrm{enterocyte}(\mathrm{IL}2)\_\mathrm{Glu}}}\right) \end{array}$$

(21) $$\begin{array}{c} \frac{{\mathrm{dA}}_{\mathrm{undissolved}(\mathrm{Co})}}{\mathrm{dt}}=\left(\left(\frac{1}{{tranist}_{\mathrm{Ce}}}\right)\times A_{\mathrm{undissolved}(\mathrm{Ce})}\right)-\left(\left(\frac{1}{{tranist}_{\mathrm{Co}}}\right)\times A_{\mathrm{undissolved}(\mathrm{Co})}\right)-(dissolution\;\times \\ A_{\mathrm{undissolved}(\mathrm{Co})}\times \left({Solubility}_{\left(\mathrm{Co}\right)}-C_{\mathrm{dissolved}(\mathrm{Co})}\right))+\left(\frac{V_{max,\beta -glucuronidase}\times C_{u,\mathrm{enterocyte}(\mathrm{Co})\_\mathrm{Glu}}}{K_{m,\beta -glucuronidase}+C_{u,\mathrm{enterocyte}(\mathrm{Co})\_\mathrm{Glu}}}\right) \end{array}$$

In the above equations, V_i_, Q_i_, C_i_, C_u,i_ A_i,_ K_p,i_, C_i_Glu_, C_u,i_Glu_, A_i_Glu,_ K_p,i_Glu_, and transit_i_ are volume of the organ, blood flow rate to the organ, GT concentration in the tissue, GT unbound concentration in the tissue, GT amount, GT tissue to plasma partition coefficient, GT-glu concentration, GT-glu unbound concentration, GT-glu amount, GT-glu tissue to plasma partition coefficient, and intestinal transit time, respectively, for organ i; k_a_ and k_a,sys_ are the first-order intestinal absorption rate constants for intestinal lumen to enterocytes and enterocytes to blood, respectively; GER is the gastric emptying rate; R_B:P_ is the blood-to-plasma ratio; f_u,p_ is the unbound fraction of a drug in plasma; PS_Li_ is the permeability-limited distribution coefficient of liver; V_max_, J_max_, V_max,β-glucuronidase_, and K_m_ are the in vivo maximum rates of reaction and Michaelis–Menten constant of microsomes, transporters, and gmGUS; RDM, RLM, and RnM are the microsomal glucuronidation of the duodenum, liver, and jejunum–ileum–colon, respectively; ISEF is the inter-system extrapolation factor for transporter activity; and Mrp3_i_, Mrp2_i_, Bcrp_i_, and Oatps_i_ are the total amount of transporters for organ i. The physiological parameters such as blood flow rates and tissue volumes were obtained from the literature and are listed in Table S1 [34,35,36,37]. Permeability-limited distribution was attributed to slowly perfused tissues and liver compartments, whereas the rest of the tissues were assumed to be “well-stirred” compartments and thus assigned a perfusion-limited distribution of GT. Permeability-limited organs were divided into two subcompartments, such as vascular and extravascular spaces, with permeability-limited distribution coefficients such as PS_sp_ and PS_li_ for slowly perfused tissues and liver compartments, respectively. Based on the literature, the percentages of vascular spaces in slowly perfused tissues and liver compartments were fixed to 6% and 21% of the total compartment volumes, respectively [34]. The intestine was divided into the duodenum, jejunum, proximal ileum, distal ileum, caecum, and colon. The lengths of various regions of intestine in the model were assigned based on regional lengths of intestine used to generate in vitro microsomal data [38]. The proximal and distal ileum were assigned lengths of 10 cm each based on the literature [39]. Other gastrointestinal tract-related physiological parameters like pH, transit time, basal fluid volumes, stomach wall volume, and enterocyte volumes were obtained from the literature and are given in Table S2 [32,36,40,41,42].

GT metabolism was defined using literature-reported in vitro Michaelis–Menten kinetic parameters generated from microsomes of rat intestine and liver [18]. The K_m_ (mM) values were taken from the literature and the in vitro maximum rates of glucuronidation in various microsomes were extrapolated to in vivo values by using the following IVIVE equation [18,43].

(22) $$\begin{array}{c} {in\;vivo\;V}_{max}={in\;vitro\;V}_{max}\left(\frac{\frac{nmol}{min}}{mg\;of\;microsomal\;protein}\right)\times MPPGL\;or\;MPPGI\;\left(\frac{\;mg\;of\;microsomal\;protein}{g\;of\;liver\;or\;intestine}\right)\times \\ organ\;wet\;weight\;(g) \end{array}$$

where MPPGL is microsomal protein per gram liver (46 mg/g), MPPGI is microsomal protein per gram of intestine (15.5 mg/g), and wet weights for different regions of the intestine and liver were obtained from the literature [40,44,45]. The IVIVE-generated in vivo V_max_ and K_m_ values representing UGT-mediated glucuronidation are given in Table S3. Since glucuronides are the major circulating metabolites of GT in rats, only UGT-mediated metabolism was considered in our model for GT metabolism in the rat liver and intestines [12,18,20,46].

To model the transporter-mediated disposition of GT-glu, the liver was divided into vascular, extravascular, and bile spaces, and intestinal segments consisted of lumen and enterocytes in the model. Transporter involvement was defined using literature-reported in vitro J_max_ and K_m_ values from various cell lines that express human or mouse transporter proteins as surrogates to the rat transporter proteins. Human UGT1A9-overexpressing HeLa cell lines were used to study the rate of GT-glu transport by the human BCRP transporter [24]. Human UGT1A1-overexpressing MDCKII-MRP2 cell lines were used to study the influence of human MRP2 transporter on flavonoid glucuronide efflux rate [47]. Human Embryonic Kidney cells (HEK) 293 cells were transfected with pBabeCMVpuro-mMrp3 to express mouse Mrp3, and these cell lines were used to study the transport of GT-glu [31]. The in vitro J_max_ and K_m_ values for the transport of GT-glu from the above cell lines were 170 pmol/mg/min and 11.3 mM, 6.86 pmol/mg/min and 1.73 mM, and 30 pmol/mg/min and 1.3 mM for BCRP, MRP2, and Mrp3, respectively. IVIVE was performed to convert these in vitro values to in vivo values by using tissue weight, ISEF, and quantitative proteomic data for respective transporter tissue abundance in rats. Since transporter abundance was not reported for the cell lines used for kinetic studies and there were likely inter-species differences in the transporter kinetics, an inter-system extrapolation factor (ISEF) was used to correct for these variations. The quantitative proteomic data were obtained from the literature that employed various analytical techniques, e.g., LC-MRM proteomics, ELISA, and Western blot (footnotes in Table S4). These abundance values were scaled to the whole organ using the equation below prior to incorporation into the model.

(23) $${Transporter}_{i}=Protein\;abundance\;\left(\frac{pmol\;or\;ng}{mg\;of\;membrane\;protein}\right)\times Membrane\;protein\;\left(\frac{mg}{g\;tissue}\right)\times Total\;organ\;wt\;(g)$$

where ${Transporter}_{i}$ is the amount of a given transporter in the whole organ expressed in pmol or ng, and protein abundance is the abundance of protein per mg of membrane protein obtained from either one of the techniques mentioned above. The membrane protein scaling factors for the liver and different regions of the intestine were obtained from the literature [48]. These scaled transporter amounts in various rat organs are given in Table S4, where the liver is shown to have high expression of Mrp2 and less Bcrp. However, Bcrp expression is more abundant in the intestine compared to Mrp2 [49,50]. It can also be observed from Table S4 that the ratio of apical to basolateral transporter expression (i.e., Bcrp + Mrp2 vs. Mrp3) is high in the duodenum and jejunum, where most of the GT-glu is effluxed into the lumen [51,52]. This ratio is low in the distal part of the intestine, suggesting higher absorption of GT-glu from this region. The relatively high expression of Mrp3 in the latter regions of the intestine also enables the systemic absorption of intestine-generated GT-glu from the gmGUS-regenerated GT. Furthermore, an intestinal perfusion study of GT also showed high luminal excretion of glycone conjugates in the duodenum and jejunum compared to the ileum and colon, which could also be related to the higher levels of apical efflux transporter expression in the upper intestine [38]. These observations necessitate the use of ACAT-like multi-compartmentalization of the intestine in our model so that region-specific enteric efflux and metabolism can be incorporated and assessed.

To capture the regeneration of GT aglycone from GT-glu metabolites by intestinal microflora, gmGUS activity was incorporated into the model at the distal ileum and colon regions of the intestine (Figure 1). Literature-reported in vitro kinetic parameters associated with gmGUS activity were used in the model, where fresh feces were collected from rat colon and incubated with the glycone metabolite [43]. The in vitro V_max_ values were converted to in vivo values by using scaling factors calculated based on protein content per gram of feces and lumen volumes of both the distal ileum and colon. The in vivo V_max_ and K_m_ values for gmGUS activity are given in Table S3.

### 2.2. Model Fitting and Parameter Estimation

Drug-related parameters such as f_u,p_, R_B:P_, intrinsic solubility, and fraction unbound in microsomes for GT and GT-glu are given in Table 1. These values were fixed during model fitting and were either obtained from the literature or predicted using the ADMET predictor [9,13,23,24,26,27,31,47,53,54]. K_p_ values that were calculated using the MATLAB (R2023b) function “calculateTissuePartition.m” were given in Table S5. This function computes K_p_ values based on the Rodgers and Rowland equation [55]. Other drug-related clearance parameters, as discussed above, were also fixed while fitting the model to in vivo PK data. To estimate a few model parameters such as permeability-limited distribution coefficients (PS_Li_), K_p,scalar_ for rapidly and slowly perfused tissues, ISEF for transporters, and oral absorption parameters (k_a_ and k_a,sys_), the model was fitted to in vivo PK data obtained from the literature. The GT PK study performed by Zhou et al. in fasted rats was used to fit the model [12]. In Zhou’s study, Sprague-Dawley rats were administered GT IV or PO at different doses. GT was solubilized in 1:1 dimethyl sulfoxide and polyethylene glycol solution for administration into the rat tail vein at a dose of 12.5 mg/kg. For oral dosing, GT suspension in 0.5% sodium carboxymethylcellulose (Na-CMC) was administered at various dose levels such as 6.25, 12.5, and 50 mg/kg. Blood samples were collected at 12 time points starting from 5 min to 36 h, and plasma was separated by centrifugation from these samples. The GT concentration in plasma (referred to as “free GT” in Zhou’s study) was determined by extraction with a mixture of methyl tert-butyl ether/pentane and HPLC with a diode array detector at a wavelength of 260 nm. The GT glucuronide concentration was determined by first incubating plasma with a glucuronidase solution for 24 h to fully convert all GT glucuronides to GT (total GT) and then subtracting “free GT” from total GT. As a result, GT glucuronide concentration included all GT glucuronide isomers with different sites of glucuronidation and is referred to as GT-glu in the current study. The plasma PK data obtained with the 12.5 mg/kg dose were used for model fitting as this dose was given by both IV and PO routes. Distribution- and excretion-related model parameters for both GT and GT-glu were estimated by using the IV data. These estimated parameters were applied to the GT oral model and the oral model was further used to estimate absorption-related parameters by fitting to the PO data from the 12.5 mg/kg dose. The intestinal absorption rate constant (k_a_) was fixed to the value obtained from the literature where the transport of genistein across rat small intestinal epithelium IEC-18 cell lines was studied [29]. On the other hand, the oral suspension-related solubility factor and stomach absorption rate constant (k_a,ST_) were estimated by fitting to the PO data.

To evaluate the model performance after parameter estimation, the newly developed PBPK model was used to simulate plasma PK and urinary excretion at a low PO dose (6.25 mg/kg) and a high PO dose (50 mg/kg), and the simulated results were compared to observed data obtained at the same doses. Furthermore, observed plasma PK data obtained at 4, 20, and 40 mg/kg PO doses from a separate study by Kwon et al. were also used for evaluating model performance [56]. All observed PK data used in the current study were obtained and digitized using WebPlotDigitizer 4.7 software to extract concentration and time data from relevant graphs of previously published studies. The effect of hepatobiliary and enteric transporters on the plasma and colonocyte GT and GT-glu concentrations was also determined by separately inhibiting liver apical efflux for the EHR pathway, intestinal apical efflux for the EER pathway, intestinal basolateral efflux for the HER pathway, and liver basolateral uptake for the HER_uptake mechanism (Figure 1).

### 2.3. Sensitivity Analysis

Parameter sensitivity analysis was performed to determine the influence of input parameters on plasma or colonocyte concentration–time profiles of GT and GT-glu. Global sensitivity analysis (GSA) was performed in MATLAB SimBiology (R2023b), where the model parameters were varied by 2 times the model value, and their effect on plasma concentration was determined. As a result, Sobol indices were generated for each tested parameter. The model input parameters with Sobol index values of more than 0.05 were selected as pivotal parameters [57]. Furthermore, the influence of these pivotal parameters on model observables, such as the area under the curve (AUC) and maximum concentration (C_max_) in plasma, was quantified.

## 3. Results

### 3.1. Parent–Metabolite Middle-Out PBPK Model Building and Parameter Estimation

**Figure 2:**
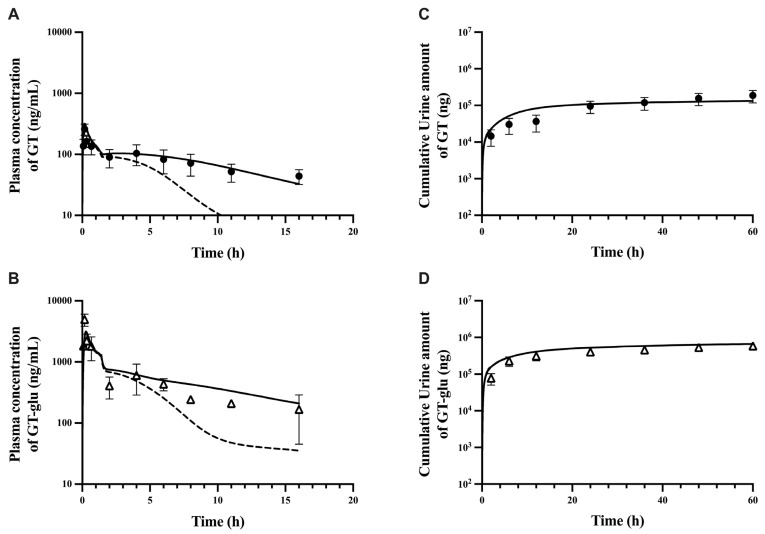
Plasma concentration (**A**,**B**) and cumulative urine amount (**C**,**D**) of genistein and its glucuronide representing model fitting after oral administration of 12.5 mg/kg of genistein. Solid lines are predicted profiles. Dashed lines are predictions without gut microbial beta-glucuronidase (gmGUS) activity. Closed circles and open triangles are observed data for genistein (**A**,**C**) and genistein glucuronide (**B**,**D**), respectively.

The rat PBPK model developed to capture the plasma concentration profiles of GT and its major circulating metabolite, GT-glu, was initially fitted to observed data obtained from the intravenous administration of GT at a dose of 12.5 mg/kg. As described above, several drug-related parameters for both GT and GT-glu were obtained from the literature and fixed to the model. Other GT distribution-related model parameters such as K_p,scalar_, PS_sp_, and PS_li_ were estimated by fitting the model to the observed data. To adequately capture the GT plasma profile after its regeneration by intestinal bacterial deglucuronidation, an in vivo solubility factor of 2 was incorporated, and a first-order absorption rate constant of 500 1/h was used for the absorption of free GT from enterocytes into portal vein circulation. Furthermore, ISEF for hepatic basolateral uptake (Oatps), basolateral (Mrp3), and apical (Mrp2 + Bcrp) efflux transporters were also estimated using intravenous GT-glu plasma PK, as both the plasma C_max_ of GT-glu and the extent of GT regeneration via intestinal deglucuronidation are transporter-dependent. The estimated and fixed model parameter values are listed in Table 2. The result of fitting the PBPK model to intravenous data, an overlay of predicted versus observed plasma PK profiles of both GT and GT-glu is given in Figure S1. In addition to comparing the overlay of observed and predicted PK profiles, a fold error or prediction error (ratio of predicted/observed values) was also calculated. The calculated fold errors for the AUC of GT and GT-glu were 1.5 and 1.1, respectively. Thus, the estimated model parameters were fixed to the model, and the plasma PK profile obtained from oral administration of 12.5 mg/kg GT was used to perform oral PBPK model fitting. While estimating oral absorption, a suspension solubility factor (Table 2) was used to allow higher solubility than literature-reported intrinsic solubility of GT as both oral formulation excipients and lipid-rich GI fluids are known to markedly alter in vivo drug solubility in the upper gastrointestinal tract (stomach and duodenum). The first-order absorption rate constant obtained from the literature was used to define the absorption of genistein in the intestine [29]. The overlays of observed and predicted plasma PK profiles are given in Figure 2A,B. The prediction error for plasma pharmacokinetic parameters such as AUC, C_max_, t_1/2_, and absolute bioavailability for total genistein was within the 2-fold range (Table 3). A good overlap of observed and predicted cumulative amount of urinary excretion of GT and GT-glu can also be seen in Figure 2C,D.

### 3.2. PBPK Model Performance

**Figure 3:**
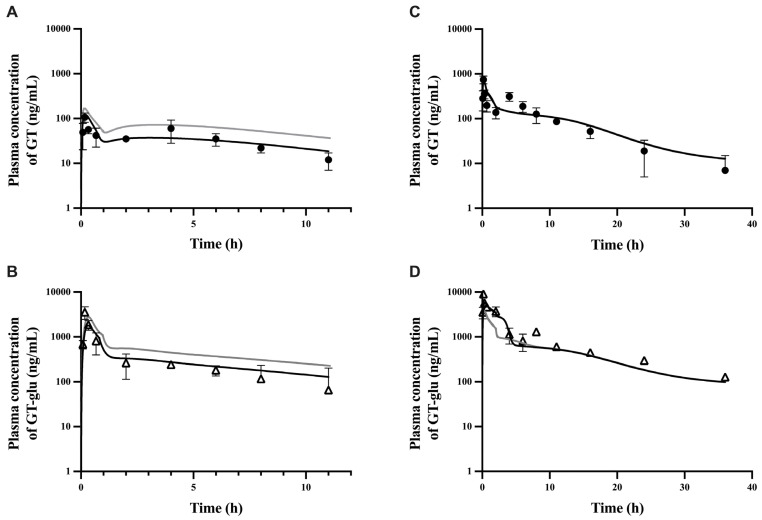
Plasma concentration of genistein and genistein glucuronide after oral administration of 6.25 mg/kg (**A**,**B**) and 50 mg/kg (**C**,**D**) genistein. Solid black lines are predicted profiles after accounting for dose-dependent solubilities obtained by either reducing the in vivo solubility factor or increased soluble fraction in jejunum at low and high doses, respectively. Solid gray lines are predictions obtained without adjustments. Closed circles and open triangles are observed data for genistein (**A**,**C**) and genistein glucuronide (**B**,**D**), respectively.

The fitted PBPK model was further evaluated by comparing predicted and observed plasma PK profiles and cumulative urinary amounts at two different dose levels. At the low dose of 6.25 mg/kg, the model moderately overpredicted both GT and GT-glu plasma levels throughout nearly all time points (gray lines in Figure 3A,B). In contrast, at the high dose of 50 mg/kg, the model did a fair job predicting both GT and GT-glu plasma levels (gray lines in Figure 3C,D), except for the noticeable underprediction of GT plasma concentration at around 5 h and GT-glu plasma concentration before 4 h. The overprediction at the low dose appears to be due to the use of an intrinsic solubility correction factor for GT in the fitted model (Table 2), as resetting this factor from 2.0 back to 1.0 resulted in substantially improved predictions for both GT and GT-glu (black lines in Figure 3A,B).

**Figure 4:**
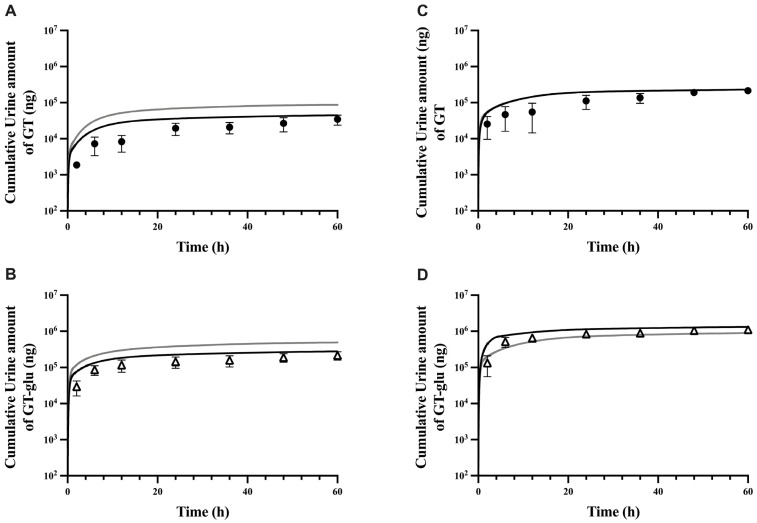
Cumulative urine amounts of genistein and genistein glucuronide after oral administration of 6.25 mg/kg (**A**,**B**) and 50 mg/kg (**C**,**D**) genistein. Solid black lines are predicted profiles after accounting for dose dependent solubilities obtained by either reducing the in vivo solubility factor or increased soluble fraction in jejunum at low and high doses, respectively. Solid gray lines are predictions obtained without adjustments. Closed circles and open triangles are observed data for genistein (**A**,**C**) and genistein glucuronide (**B**,**D**), respectively.

To improve prediction at the high dose, a moderated suspension solubility factor of 10 was applied to the jejunum to allow greater in vivo GT solubility in that compartment, in addition to the stomach and duodenum (Table 2). As a result, the use of this suspension solubility factor in the upper gastrointestinal tract markedly improved the prediction for GT-glu in the early hours (before 4 h) at the high dose, while having a negligible effect on the GT plasma concentration (black lines in Figure 3C,D), likely due to the rapid conversion of absorbed GT to GT-glu in the enterocytes and hepatocytes. After this fine-tuning of the fitted model, the fold errors of pharmacokinetic parameters at all dose levels were within a 2-fold error (Table 3), and the predicted cumulative urinary excretion of GT and GT-glu also improved (Figure 4). A final evaluation of the model performance was performed using an independent study by Kwon et al. [56], where male Sprague-Dawley rats were given oral doses of GT suspension in 0.2% Na-CMC at three different dose levels: 4, 20, and 40 mg/kg. Plasma concentration profiles in this study were given as total genistein levels, i.e., free genistein and glucuronidated genistein. Therefore, the total plasma genistein levels simulated by the model were compared to the literature values (Figure S2), and fold error ranges for AUC and C_max_ values at all the dose levels were 0.9–1.3 and 0.9–1.1, respectively. Non-linear pharmacokinetics was previously observed where biliary and urinary excretion of total genistein showed nonproportionality with increased dose levels [12]. Based on the final overlay results at all doses, our model was able to capture non-linearity in the GT PK. The verified model was also used to simulate the impact of gmGUS activity on the plasma half-lives and AUC of GT and GT-glu. In the absence of gmGUS, the plasma half-lives of GT and GT-glu were reduced by 3-fold and 4-fold, respectively, and their AUC values decreased by 2-fold (Figure 2A,B).

### 3.3. Model Sensitivity Evaluation

By performing GSA, where a given set of parameters are varied simultaneously to evaluate the relative impact of each parameter, it was evident that genistein solubility was the most crucial factor influencing the plasma C_max_ and AUC of GT, whereas transporter activities were more influential for the plasma C_max_ and AUC of GT-glu (Figure S3). When the solubility of genistein was varied by 2-fold, the AUC and C_max_ of GT varied by about 4-fold and 2-fold, respectively (Figure S5). The influence of transporter activity on GT-glu plasma profiles shows that plasma C_max_ values were highly dependent on ISEF_Mrp3_ and ISEF_apical_ transporters (Table 2), with nearly 3-fold variations in the tested range (Figure S4B,C). On the other hand, ISEF_uptake_ transporter activity shows a similar effect on the plasma AUC and C_max_ of GT-glu, albeit its overall influence on PK profiles is smaller than the other two transporter factors (Figure S4D). When the influence of intestinal glucuronidation was compared with hepatic glucuronidation, the sensitivity analysis results showed that duodenal glucuronidation has a higher influence on the GT-glu plasma AUC than hepatic glucuronidation, whereas both have an equal influence on the GT-glu plasma C_max_ (Figure S6A). The second peak observed in the GT plasma profile at about 4 h was largely due to glucuronide recycling in the intestine as a greater absorption of regenerated GT in the caecum helped to produce the second peak (Figure S5D). In addition to the above-discussed sensitivities, GT-glu renal excretion (Figure S4A) and colon glucuronidation (Figure S5C) also moderately influence the GT and GT-glu plasma profiles, respectively.

### 3.4. Transporter-Dependent Enterohepatic Recirculation of GT and GT-Glu

**Figure 5:**
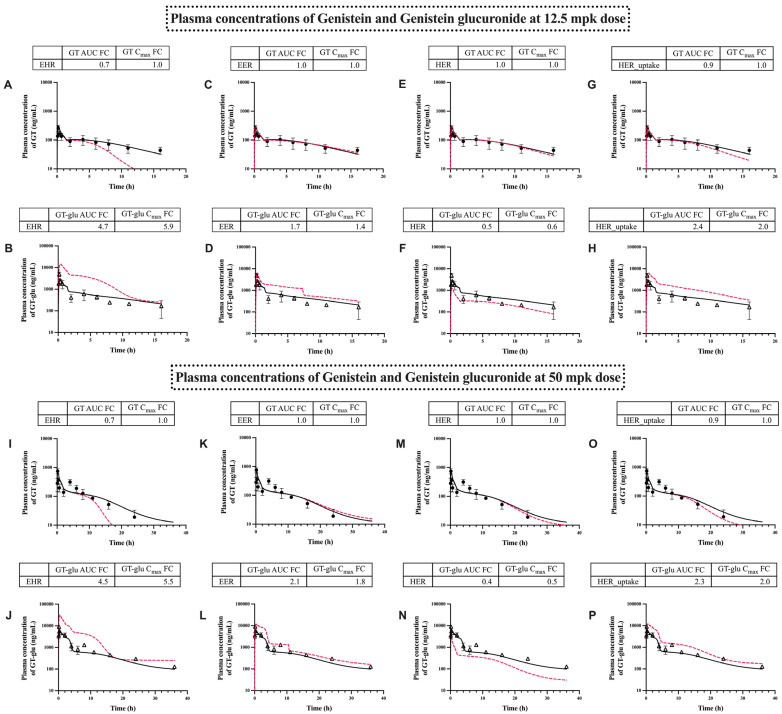
The influence of recycling mechanisms on the plasma concentrations of genistein and genistein glucuronide after the oral administration of 12.5 mg/kg (**A**–**H**) and 50 mg/kg (**I**–**P**) of genistein. The graphs (**A**,**B**,**I**,**J**) represent the effect of EHR on the plasma concentrations of genistein and its glucuronide at 12.5 mg/kg and 50 mg/kg doses, respectively. The graphs (**C**,**D**,**K**,**L**) represent the effect of EER on the plasma concentrations of genistein and its glucuronide at 12.5 mg/kg and 50 mg/kg doses, respectively. The graphs (**E**,**F**,**M**,**N**) represent the effect of HER on the plasma concentrations of genistein and its glucuronide at 12.5 mg/kg and 50 mg/kg doses, respectively. The graphs (**G**,**H**,**O**,**P**) represent the effect of HER_uptake on the plasma concentrations of genistein and its glucuronide at 12.5 mg/kg and 50 mg/kg doses, respectively. The solid black lines are model predictions. The red-colored dashed lines are model predictions obtained by disabling the respective mechanisms. The closed circles and open triangles are the observed genistein and genistein glucuronide data, respectively. Fold change (FC) = without recycling mechanism/with recycling mechanism.

The simulation results for various aforementioned GT-glu recycling pathways at the 12.5 mg/kg and 50 mg/kg doses are shown in Figure 5, where the impact of the individual glucuronide recycling pathway was assessed by selectively disabling the relevant transporter(s) responsible for the recycling pathway in our model.

Specifically, biliary efflux transporters (hepatic Mrp2/Bcrp) were disabled for assessing the enterohepatic recycling (EHR) pathway; enteric basolateral effux transporter (enteric Mrp3) was disabled for assessing the hepatoenteric recycling (HER) pathway; enteric luminal efflux transporters (enteric Mrp2/Bcrp) were disabled for assessing the enteroenteric recycling (EER) pathway; and lastly, hepatic sinusoidal uptake transporter (hepatic Oatps) was disabled for assessing the hepatic uptake mechanism (HER_uptake), which helps enable the HER pathway by uptaking intestine-generated glucuronides into the hepatocytes. Among the four recycling pathways examined, EHR involving the biliary excretion of glucuronide shows the highest fold change in AUC values for GT and GT-glu at both dose levels (Figure 5A,B,I,J). The second most impactful recycling mechanism was the HER pathway, either involving the basolateral efflux of intestine-generated GT-glu into the portal vein (Figure 5E,F,M,N) or the sinusoidal uptake (HER_uptake) of intestine-generated GT-glu into the hepatocytes (Figure 5G,H,O,P), with about 2-fold changes in both GT-glu plasma AUC and C_max_. Additionally, disabling intestinal luminal efflux (EER pathway) demonstrated a significant increase in the GT-glu plasma concentration (Figure 5C,D,K,L), as intestine-generated glucuronides shift from luminal efflux to basolateral efflux into the portal vein. It also appears that this effect became more pronounced at the higher dose (Figure 5L vs. Figure 5D). Based on the sensitivity results (Figure S3), the effects of these transporters were expected to be minimal on the GT profiles. However, disabling EHR (biliary efflux transporters) also influenced the AUC and half-life of GT (Figure 5A,I), likely due to reduced enterohepatic recirculation of GT-glu. In addition, GT plasma AUC and half-life were moderately influenced by disabling HER_uptake (sinusoidal uptake transporters), reducing its residence time.

Furthermore, the effect of these transporter-dependent recycling mechanisms on local tissue exposure in colonocytes is shown in Figure S7. Disabling the intestinal transporter-related EER or HER pathway (Figure S7D,F,L,N) clearly showed a large impact on the local colonocyte exposure of GT-glu by increasing the tissue AUC and C_max_ by about 2-fold. On the contrary, disabling the liver transporter-related EHR or HER_uptake pathway decreased the local colonocyte AUC of GT-glu but did not affect its C_max_ in the colonocytes (Figure S7B,H,J,P). It is noteworthy that these effects observed in the colonocytes (Figure S7) were opposite to the effects observed in the plasma (Figure 5) for all recycling pathways examined, except for the intestinal luminal efflux EER pathway.

### 3.5. The Effect of gmGUS on the Plasma and Enterocyte Concentration of GT

**Figure 6:**
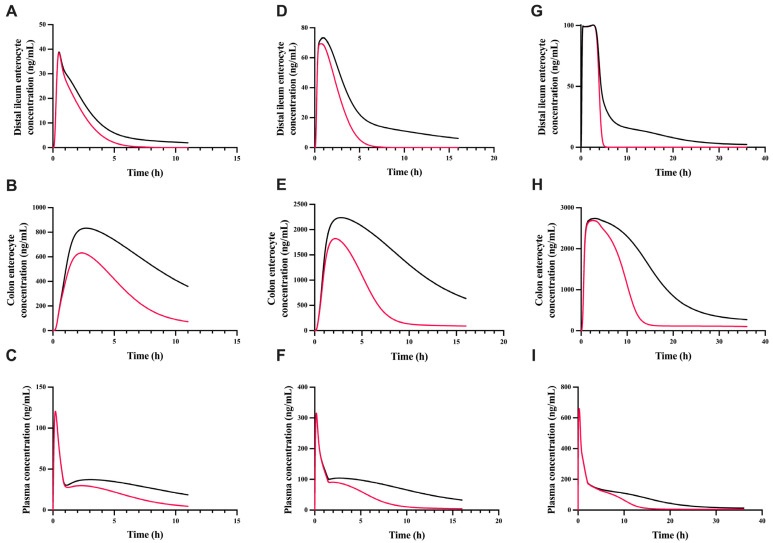
Impact of gut microbial glucuronidase (gmGUS) on genistein exposure in enterocytes and plasma after oral administration of genistein at 6.25 mg/kg (**A**–**C**), 12.5 mg/kg (**D**–**F**), and 50 mg/kg (**G**–**I**) doses. Genistein exposure in enterocytes of distal ileum across dose levels is shown in Figures (**A**,**D**,**G**). Genistein exposure in enterocytes of colon across dose levels is shown in Figures (**B**,**E**,**H**). Genistein plasma concentrations across dose levels were are in Figures (**C**,**F**,**I**). Black solid lines are model-predicted profiles with glucuronidase activity. Red solid lines are model predictions without glucuronidase activity.

To assess the effect of gmGUS on the plasma and enterocyte exposure of GT, gmGUS activity was set to zero to simulate the condition without gmGUS activity at different oral doses (Figure 6). The presence of gmGUS enzymes prolonged the residence time and increased the plasma AUC of the parent compound by approximately 3-fold and 2-fold, respectively (Figure 6C,F,I). It also increased the GT enterocyte AUC by 1.4- to 2.1-fold in the distal ileum and colon across the tested dose levels. The colonocyte C_max_ of GT demonstrated a notable difference in the presence and absence of gmGUS activity, particularly at the two lower doses (Figure 6B,E,H). In addition, GT concentrations in colonocytes were markedly higher than those in the distal ileum and plasma. This is likely due to the high gmGUS activity in the distal ileum and colon (Table S3), enabling the rapid conversion of GT-glu to GT in the lumen and the subsequent absorption of GT into the colonocytes.

## 4. Discussion

In this study, we developed and evaluated a new rat parent–metabolite middle-out PBPK model for GT and GT-glu by incorporating relevant mechanistic processes for GT and GT-glu, including hepatic and intestinal glucuronidation of GT, transporter-mediated hepatobiliary and enteroluminal disposition of GT-glu, and the gmGUS-dependent conversion of GT-glu back to GT. This approach enabled us to assess the roles of intestinal and hepatobiliary metabolism and transport in the disposition of a parent drug, GT, and its glucuronide metabolite, GT-glu. Our model is distinct from previously reported PBPK models of GT/GT-glu in our emphasis on the transporter-mediated disposition of GT-glu and the impact of various recycling pathways on the plasma and local colonocyte PK profiles of GT and GT-glu [21,22,23,24].

To evaluate the performance of our parent–metabolite middle-out PBPK model, we tested the predictions of GT and GT-glu plasma concentrations and the cumulative amounts excreted in urine following the oral administration of GT at two different dose levels. The plasma PK predictions obtained from oral dosing appeared to be highly sensitive to GT solubility, which is consistent with its well-known poor aqueous solubility [8] (Figure 3, Figure 4, Figures S3 and S5A). During the oral dose model’s development, a suspension solubility factor (Table 2) had to be included to account for the much higher in vivo solubility of GT in the presence of formulation excipients (i.e., 0.2–0.4% Na-CMC), lipid-rich GI fluids, and/or supersaturation in the stomach and duodenum in order to match the very rapid appearance of GT and GT-glu in the plasma (T_max_ at 10 min for both GT and GT-glu; Figure 2A,B) after oral gavage in rats. Similar difficulty in the model fitting of this very rapid appearance of GT and GT-glu in the plasma after oral dosing was also reported by Schlosser et al. [23]. The importance of GT solubility was further demonstrated in our attempt to correct the underprediction in the early hours (<4 h) after the high oral dosing (50 mg/kg) (Figure 3D). Here, a slightly moderated suspension solubility factor (10 instead of 36; Table 2) was applied to jejunum at the high PO dose and it resulted in a markedly improved match between the model simulation and observed GT-glu plasma profiles (Figure 3D). However, the match between model-predicted and observed GT plasma profiles did not improve simultaneously (Figure 3C). Although PK predictions for oral administration at all dose levels fell within the acceptable FE range of 0.5 to 2.0, there remained a slight underprediction at the first C_max_ value in the GT-glu simulations (Table 3). The reason behind this is not entirely clear, but the ratio of GT-glu to GT at this time point seemed to be disproportionately high compared to nearby time points, and thus, our model is unable to capture this particular time point.

Generally, the liver is considered the primary organ for UGT-mediated glucuronidation; however, some studies on phenolic compounds and GT microsomal data suggest a significant contribution of intestinal glucuronidation after oral administration [8,9]. Our model’s sensitivity analysis results showed that the duodenal metabolism could play a significant role in the generation of glucuronide metabolites (Figure S6). Even though the in vitro microsomal results suggest that jejunal microsomes have a greater capacity to glucuronidate genistein (Table S3), model sensitivity analysis revealed little impact from jejunal glucuronidation on the plasma AUC and C_max_ of GT-glu (Figure S6A). This could be due to the higher fraction absorbed (Fa) in the duodenum (0.25) compared to the jejunum (0.1) predicted by our model and further supported by the very rapid plasma C_max_ observed within 10 min (Figure 2A,B and Figure 3A,B), indicating very rapid absorption in the stomach and duodenum. To assess the impact of jejunal metabolism, our PBPK model was modified to mimic intrajejunal dosing. Sensitivity analysis of this intrajejunal-dosed model revealed a substantially increased impact of jejunal glucuronidation on the plasma AUC and C_max_ of GT-glu (Figure S6B), as jejunal dosing would greatly increase the amount of genistein that reaches the jejunum and is available for jejunal glucuronidation, in contrast to intragastric dosing by oral gavage, where most of the genistein has been absorbed before reaching the jejunum.

Hepatobiliary and enteric transporters differentially affected GT and GT-glu plasma and colonocyte exposure, as revealed by disabling individual pathways in our model (Figure 5 and Figure S7). Although these transporters preferentially affected the plasma and colonocyte exposure of GT-glu, they could still influence those of GT. For example, disabling EHR (biliary efflux transporters) reduced the plasma and colonocyte AUC and half-life of GT (Figure 5A,I and Figure S7A,I), likely due to the reduced biliary excretion of GT-glu (Figure S8) and subsequent reduction in the enterohepatic recirculation of GT-glu. Furthermore, for GT-glu, the effects of transporters observed in the colonocytes (Figure S7) were opposite to the effects observed in the plasma (Figure 5) for all recycling pathways examined, except for the intestinal luminal efflux EER pathway. This result challenges the conventional practice of using plasma exposure to represent local tissue exposure, emphasizing the importance of measuring local exposure and/or assessing local exposure using PBPK model-based approaches.

The regeneration of aglycones by gmGUS in the intestine can play a critical role in extending plasma half-lives and increasing drug exposure in enterocytes. Simulations using our model demonstrated up to 2-fold-greater plasma and enterocyte exposures for GT when gmGUS activity was present (Figure 6). The dose-dependent effect on the colonocyte C_max_ of GT from these simulations could be due to several possible reasons, e.g., the saturation of biliary efflux transporters, saturation of gmGUS, and/or solubility-limited GT concentration in the colon lumen at the high dose [12]. To identify the likely reason, a global sensitivity analysis of the model was performed for colonocyte GT and GT-glu exposures (AUC and C_max_), and the results revealed that the colonocyte GT exposure was most affected by GT’s intrinsic solubility, whereas biliary efflux transporters and gmGUS had much less impact (Figure S9A,C). In contrast, hepatobiliary transporters, colonocyte transporters, and colonocyte glucuronidation had the greatest effect on the colonocyte GT-glu exposure (Figure S9B,D). To further verify these discoveries, model simulations showed that simulated GT-glu liver extravascular concentrations (maximal 60 ng/mL at 50 mg/kg PO dose; Figure S10A) would be well below the K_m_ values of biliary efflux transporters from surrogate species (468 and 3054 ng/mL for Mrp2 and Bcrp, respectively; Table 1), indicating a low possibility for GT-glu to saturate biliary efflux transporters at the high dose. In addition, simulated GT-glu concentrations in the lumens of the distal ileum, caecum, and colon where gmGUS activities were present (maximal 0.6 ng/mL; Figure S10F–H) were also well below the K_m_ value of gmGUS (822 ng/mL; Table S3), indicating a low possibility for GT-glu to saturate gmGUS at the high dose. In comparison, simulated dissolved GT concentrations in the lumens of the distal ileum, caecum, and colon (Figure S11D–F) showed a pronounced dose dependency limited by the in vivo solubility of GT, which was estimated using the corrected GT intrinsic solubility (48,000 ng/mL or 2 × intrinsic solubility; Table 1 and Table 2). Taken together, it was likely the limited in vivo GT solubility in the lower GI lumens, rather than the saturated biliary efflux or gmGUS, that caused the dose-dependent effect on the colonocyte GT exposure (Figure 6). The simulated high local exposure in the colonocytes (Figure 6B,E,H) would raise concerns about potential drug-induced intestinal toxicity due to high levels of regenerated aglycone accumulation in the gut for compounds such as SN-38, morphine, mycophenolic acid, etc. [6]. Given the lack of correlation between the simulated plasma and colonocyte concentrations (Figure 6), this further illustrates the importance of measuring local exposure and/or assessing local exposure using PBPK model-based approaches to understand local toxicity or improve local therapeutic effect, e.g., inflammatory bowel disease and colon cancer [58,59].

Our current model has several major limitations. First, it was not built bottom-up using rat-specific kinetic data for relevant transporters, and ISEFs for transporters were used during IVIVE and model fitting. This is primarily due to the lack of rat transporter data in the literature at the moment, such as in vitro kinetics for relevant rat transporters and transporter protein abundances in in vitro systems used to measure uptake/efflux kinetics. As a result, it is currently untenable to build a fully mechanistic IVIVE-based PBPK model for GT and GT-glu in rats. Second, the use of solubility correction factors (Table 2) increases model uncertainty. Indeed, as the model sensitivity analysis revealed (Figure S3), GT solubility has the greatest influence on the GT plasma exposure among all parameters examined as it determines the effective GT concentrations in the gastrointestinal lumens that drive its passive absorption into the tissues. However, intrinsic aqueous GT solubility is very poor (Table 1) and in vivo GT solubility in the gastrointestinal tract would be greatly affected by the presence of formulation excipients and lipids (e.g., bile acids). As such, it necessitates some forms of correction to the intrinsic solubility until actual in vivo solubility is experimentally determined. Third, model fitting and simulations did not fully recapitulate the observed PK profiles. As mentioned above, the most difficult part of model fitting was to capture the very rapid appearance of GT and GT-glu after oral dosing with T_max_ around 10 min, which was shared by Schlosser et al. in their attempt to build a PBPK model for GT and GT-glu [23]. This led to consistent underestimation of C_max_ for GT-glu (Figure 2B and Figure 3B,D). Another difficulty in the model fitting was to capture the secondary peak in the PK profiles. The observed PK data used in our study presented some challenges, such as the inconsistent timing of second peaks at different doses (Figure 2B vs. Figure 3B,D) and between GT and GT-glu for the same dose (Figure 3C vs. Figure 3D). Although better observed PK data could help, this would not rule out the possibility that our model did not fully capture mechanistic processes related to the disposition of GT and GT-glu. For example, our model lumped all GT glucuronides into a single species GT-glu and ignored the contribution of GT sulfates to the overall disposition. Future work should consider these factors once in vitro sulfate conjugation kinetics and glucuronide isoform-specific kinetics become available. Despite these limitations, our model serves as a useful modeling tool to assess the relative impact of individual mechanistic pathways (e.g., transporters and gmGUS; Figure 5, Figures S4–S6 and S8) and compare systemic and local tissue exposures (e.g., plasma vs. enterocytes; Figure 6 and Figure S7).

## 5. Conclusions

In conclusion, our parent–metabolite middle-out PBPK model for GT and GT-glu in rats incorporated hepatic and intestinal glucuronidation, transporter-mediated hepatobiliary and enteroluminal disposition, and gmGUS-dependent deglucuronidation, and it was able to achieve a reasonably good fit to the observed plasma PK profiles of GT and GT-glu at different dose levels. Our study underlies the importance of glucuronide recycling in determining local drug concentrations in the intestine and provides a preliminary modeling tool to assess the influence of transporter-mediated drug–drug interactions on glucuronide recycling and local drug exposure that are often misrepresented by systemic plasma concentrations.

## Figures and Tables

**Table 2:** Estimated model parameters attained by simultaneously fitting to genistein and its glucuronide plasma PK profiles obtained from IV and PO dosing of 12.5 mg/kg of genistein.

Parameter	Estimated Values	Standard Error	% CV	90% CI Range	Notes
Genistein					
K_p,scalar_	0.052	0.029	56%	0.014–0.103	Only applied to rapidly and slowly perfused tissues
PS_li_ (mL/h)	3.47 × 10^5^	1.74 × 10^5^	50%	1.17 × 10^5^–6.48 × 10^5^	
PS_sp_ (mL/h)	5.00 × 10^4^	3.09 × 10^4^	62%	8.67 × 10^3^–1.04 × 10^5^	
Suspension solubility factor	36.15	10.45	29%	18.08–54.28	Used for all PO in stomach and duodenum lumens; 10 for jejunum for high PO
k_a,ST_ (1/h)	0.69	0.18	26%	0.38–1.01	
F_renal_	100	Fixed	NA	NA	
k_a,sys_ (1/h)	500	Fixed	NA	NA	
Intrinsic solubility correction factor	2.0	Fixed	NA	NA	Used for IV and mid- and high PO in all GI lumens
Genistein glucuronide					
ISEF_Mrp3_	8.53 × 10^5^	4.02 × 10^5^	47%	3.16 × 10^5^–1.00 × 10^6^	
ISEF_apical_ (Mrp2+Bcrp)	8.65 × 10^5^	6.26 × 10^5^	72%	2.88 × 10^4^–1.70 × 10^6^	
ISEF_Uptake_ in liver	9.35 × 10^3^	4.45 × 10^3^	48%	3.40 × 10^3^–1.00 × 10^4^	
K_p,scalar_Glu_	0.01	Fixed	NA	NA	

NA, not applicable.

**Table 3:** Comparison of PBPK model-predicted and observed pharmacokinetic parameters of genistein and its glucuronide obtained at various dose levels of orally administered genistein.

Plasma Pharmacokinetics	Predicted (GT)	Observed (GT)	FE ^a^	Predicted (GT-Glu)	Observed (GT-Glu)	FE ^a^
6.25 mg/kg						
C_max_ (ng/mL)	120	107	1.1	1804	3568	0.5
t_1/2_ (hour)	5.5	3.2	1.7	6.2	3.4	1.8
AUC_0 to last_ (ng.h/mL)	372	388	1.0	3455	3354	1.0
%F	13%	16%	0.8	NA	NA	NA
12.5 mg/kg						
C_max_ (ng/mL)	315	260	1.2	3132	4941	0.6
t_1/2_ (hour)	10.5	9.7	1.1	13.2	15.0	0.9
AUC_0 to last_ (ng.h/mL)	1318	1228	1.1	8989	7523	1.2
%F	22%	26%	0.9	NA	NA	NA
50 mg/kg						
C_max_ (ng/mL)	659	747	0.9	5841	9050	0.6
t_1/2_ (hour)	10.4	6.7	1.5	13.0	11.3	1.2
AUC_0 to last_ (ng.h/mL)	3185	2841	1.1	25,810	29,324	0.9
%F	13%	15%	0.8	NA	NA	NA

^a^ FE, fold error (predicted value/observed value).

## Data Availability

The data presented in this study were derived from the following resources available in the public domain: https://doi.org/10.1021/jf801051d and https://doi.org/10.1016/j.ijpharm.2006.12.046.

## Supplementary Materials

The following supporting information can be downloaded at: https://www.mdpi.com/article/10.3390/pharmaceutics17070814/s1. Table S1. Rat physiological parameters for genistein PBPK models; Table S2. Rat intestinal sub compartment physiological volumes; Table S3. Final rat UGT mediated glucuronidation and intestinal bacteria mediated deglucuronidation values used in the PBPK model; Table S4. Total amount of transporters in rat organs calculated based on abundance data obtained from LC-MS/MS quantitative proteomics, western blot and ELISA techniques; Table S5. Predicted rat tissue partition coefficient values; Figure S1. Plasma concentration of genistein (A) and its glucuronide (B) representing model fitting after intravenous administration of 12.5 mg/kg genistein. Solid lines are predicted profiles. Closed circles and open triangles are observed data for genistein (A) and genistein glucuronide (B), respectively; Figure S2. Plasma concentration of total genistein concentration after oral administration of 4 mg/kg (A), 20 mg/kg (B) and 40 mg/kg (C) genistein. (D) a FE = Fold error (predicted value/observed value) 0.5 ≤ FE ≤ 2.0. Solid lines are predicted profiles. Open diamonds, open squares and asterisk are observed total genistein concentrations for 4, 20 and 40 mg/kg, respectively; Figure S3. GSA Sobol indices showing sensitivities of GT and GT-glu plasma AUC and Cmax towards model parameters. Lines with blue dots and orange dots are first order and total order Sobol indices, respectively; Figure S4. Sensitivity of genistein glucuronide plasma concentration towards (A) renal excretion of glucuronide, (B) ISEF_apical, (C) ISEF_Mrp3 and (D) ISEF_uptake. Fold change (FC) = variation in PK parameter value within 2-fold range; Figure S5. Sensitivity of genistein plasma concentration towards (A) solubility of genistein, (B) liver metabolism, (C) colon metabolism and (D) absorption of regenerated genistein in caecum. Fold change (FC) = variation in PK parameter value within 2-fold range; Figure S6. GSA Sobol indices showing sensitivity of plasma AUC and Cmax of genistein glucuronide (GT-glu) towards liver and intestinal metabolism after oral administration of 12.5 mg/kg genistein. (A) PO dosing, (B) Jejunal dosing, and lines with blue dots and orange dots are first order and total order Sobol indices, respectively; Figure S7. Influence of recycling mechanisms on colonocyte concentrations of genistein and genistein glucuronide after oral administration of 12.5 mg/kg (A–H) and 50 mg/kg (I–P) of genistein. The graphs (A,B) and (I,J) represent the effect of EHR on colonocyte concentrations of genistein and its glucuronide at 12.5 mg/kg and 50 mg/kg doses, respectively. The graphs (C,D) and (K,L) represent the effect of EER on colonocyte concentrations of genistein and its glucuronide at 12.5 mg/kg and 50 mg/kg doses, respectively. The graphs (E,F) and (M,N) represent the effect of HER on colonocyte concentrations of genistein and its glucuronide at 12.5 mg/kg and 50 mg/kg doses, respectively. The graphs (E,F) and (O,P) represent the effect of HER_uptake on colonocyte concentrations of genistein and its glucuronide at 12.5 mg/kg and 50 mg/kg doses, respectively. Solid lines are predicted profiles and dashed lines are predictions without the listed mechanisms. Black and blue colors represent genistein and genistein glucuronide, respectively. Fold change (FC) = without recycling mechanism/with recycling mechanism; Figure S8. Biliary profiles of genistein glucuronide with 10-fold decreased apical efflux transporter activity (red lines) and original values (black lines) activity; Figure S9. GSA Sobol indices showing sensitivity of colonocyte GT (A,C) and GT-glu (B,D) AUC (A,B) and Cmax (C,D) towards various model parameters after oral administration of genistein. Lines with blue dots and orange dots are first order and total order Sobol indices, respectively; Figure S10. Simulated GT-glu concentrations in the (A) liver extravascular, (B) bile, and lumens of (C) duodenum, (D) jejunum, (E) proximal ileum, (F) distal ileum, (G) caecum, and (H) colon after oral administration of 12.5 mg/kg (green lines) or 50 mg/kg (violet lines) of genistein; Figure S11. Simulated dissolved GT concentrations in the lumens of (A) duodenum, (B) jejunum, (C) proximal ileum, (D) distal ileum, (E) caecum, and (F) colon after oral administration of 12.5 mg/kg (green lines) or 50 mg/kg (violet lines) of genistein. References [60,61,62,63,64,65,66] are cited in the Supplementary Materials.

## Abbreviations

AUC, area under the curve; Bcrp, breast cancer-resistant protein; EER, enteroenteric recycling; EHR, enterohepatic recycling; f_u,p_, unbound fraction of drug in plasma; GER, gastric emptying rate; gmGUS, gut microbial β-glucuronidase; GSA, global sensitivity analysis; HER, hepatoenteric recycling; ISEF, inter-system extrapolation factor; IVIVE, in vitro-to-in vivo extrapolation; J_max_, maximum transport rate; K_m_, Michaelis–Menten constant; K_p_, tissue partition coefficient; Log P, octanol-water partition coefficient; Mrp, multidrug resistance-associated protein; MPPGL, microsomal protein per gram of liver; MPPGI, microsomal protein per gram of intestine; Oatps, organic anion transporting polypeptides; ODEs, Ordinary Differential Equations; PBPK, physiologically based pharmacokinetic; PK: pharmacokinetics; PS_Li_, permeability-limited distribution coefficient of liver; R_B:P_, blood-to-plasma ratio; RCM, rat colon microsomes; RDM, rat duodenum microsomes; RJM, rat jejunum microsomes; RLM, rat liver microsomes; RnM, rat microsomes from jejunum–ileum–colon; UGT, Uridine 5′-diphospho-glucuronosyltransferase; V_max_, maximum enzymatic reaction rate; V_max,β-glucuronidase_, maximum gmGUS hydrolysis rate.
