# Supplementary material for: A Parent–Metabolite Middle-Out PBPK Model for Genistein and Its Glucuronide Metabolite in Rats: Integrating Liver and Enteric Metabolism with Hepatobiliary and Enteroluminal Transport to Assess Glucuronide Recycling
Source: Pharmaceutics. 2025 Jun 23;17(7):814. doi: 10.3390/pharmaceutics17070814 (PMC12300792; doi:10.3390/pharmaceutics17070814)
Supplement: Supplementary file 1 [file pharmaceutics-17-00814-s001.zip › pharmaceutics-3610958-supplementary.pdf]

**A Parent-Metabolite Middle-Out PBPK Model for Genistein and its Glucuronide Metabolite in Rats: Integrating Liver and Enteric Metabolism with Hepatobiliary and Enteroluminal Transport to Assess Glucuronide Recycling**

Bhargavi Srija Ramisetty, Rashim Singh, Ming Hu and Michael Zhuo Wang

## List of abbreviations

AUC, area under the curve; Bcrp, breast cancer resistant protein; $C_{max}$, maximum concentration; Co, colon; EER, enteroenteric recycling; EHR, enterohepatic recycling; $f_{u,p}$, unbound fraction of drug in plasma; $f_{u,p}$, unbound fraction of drug in plasma; $f_{u,p,Glu}$, unbound fraction of glucuronide in plasma; $F_{renal}$, drug renal excretion factor to account for non-GFR mechanisms; $F_{renal,Glu}$, glucuronide renal excretion factor to account for non-GFR mechanisms; GER, gastric emptying rate; gmGUS, gut microbial $\beta$-glucuronidase; GSA, global sensitivity analysis; HER, hepatoenteric recycling; ISEF, Inter-System Extrapolation Factor; ISEF_apical, ISEF for Bcrp+Mrp2 in the apical membrane; ISEF_uptake, ISEF for liver Oatps in the sinusoidal membrane IVIVE, in vitro-to-in vivo extrapolation; $J_{max}$, maximum transport rate; ka_LI, absorption rate constant in large intestine; $K_m$, Michaelis-Menten constant; $K_p$, tissue partition coefficient; Li, liver; Log P, octanol-water partition coefficient; MPPGI, microsomal protein per gram intestine; MPPGL, microsomal protein per gram liver; Mrp, multidrug resistance-associated protein; Oatps, organic anion transporting polypeptides; ODEs, Ordinary Differential Equations; PBPK, physiologically based pharmacokinetic; PK, pharmacokinetics; $PS_{Li}$, permeability limited distribution coefficient of liver; $R_{B:P}$, blood to plasma ratio; RDM, rat duodenum microsome; RJM, rat jejunal microsome; RLM, rat liver microsome; R&R equation, Rodgers and Rowland equation; UGT, Uridine 5'-diphospho-glucuronosyltransferase; $V_{max}$, maximum enzymatic reaction rate; $V_{max,\beta\text{-glucuronidase}}$, maximum gmGUS hydrolysis rate.

## Supplementary Tables

### Table S1. Rat physiological parameters for genistein PBPK models

| Organ | Volume (mL)[a] | Blood flow rate (mL/h)[a] |
|---|---|---|
| Arterial blood | 3.38[b] | -- |
| Venous blood | 10.13[b] | -- |
| Kidney | 1.83 | 934 |
| Liver | 9.15 | 159 |
| Lung | 1.25 | 6624 |
| Stomach | 1.15 | 96.9 |
| Slowly perfused tissues | 185.30 | 3020.5 |
| Rapidly perfused tissues | 26.80 | 1563.3 |
| Intestine | 11.25[c] | 830.5 |
| Bile | 0.04[d] | 0.9[b] |
| Glomerular filtration rate | -- | 78.6[b] |

[a]Brown, et al., 1997 [1]; [b]davies and morris, 1993 [2]; [c]McConnell et al., 2008 [3] [d]Blouin et al., 1977 [4]

### Table S2. Rat intestinal sub compartment physiological volumes

| Organ | Basal fluid volume (mL)[a] | Stomach wall/Enterocyte volume (mL)[b] | pH[e] | Length (cm)[g] | GER/Transit time (h)[h] |
|---|---|---|---|---|---|
| Stomach | 1.80[c] | 1.0[d] | 3.9 | 10 | 0.25[e] |
| Duodenum | 0.18 | 0.29 | 5.89 | 87 | 0.15 |
| Jejunum | 0.34 | 2.53 | 6.13 | 10 | 1.35 |
| Ileum | 0.49 | 0.58 | 5.93 | 20 | 0.30 |
| Caecum | 1.21 | 0.24 | 6.58 | 5[f] | 4.29[e] |
| Colon | 0.31 | | 6.23 | 9[f] | 12.66[i] |

[a]McConnell et al., 2008 [3]; [b]Musther et al., 2017 [5]; [c]Peters, 2008 [6]; [d]Fixed; [e]Gastroplus v9.8.3; [f]DeSesso and Jacobson, 2001 [7]; [g]Lengths of each intestinal compartment was calculated based on total length of intestine obatained from Musther et al., 2017 [5]; [h]Calculated using total transit time and lengths of each compartment; [i]Kirman et al., 2012 [8]

**Table S3. Final rat UGT mediated glucuronidation and intestinal bacteria mediated deglucuronidation values used in the PBPK model**

| Microsome/Enzyme | in vivo $V_{max}$ (ng/h) | $K_m$ (ng/mL) |
|---|---|---|
| Rat duodenal microsome[a] | 4.32E+05 | 1092 |
| Rat jejunal microsome[a] | 8.61E+06 | 1791 |
| Rat ileal microsome[a] | 7.87E+05 | 2227 |
| Rat colon microsome[a] | 7.08E+04 | 861 |
| Rat liver microsome[a] | 2.53E+07 | 6378 |
| β-glucuronidase activity in distal ileum[b] | 1.12E+08 | 822 |
| β-glucuronidase activity in colon[b] | 2.20E+08 | |

[a]Chen et al., 2005 [9]; [b]Ebuzoeme, C., et al., 2021 [10]

**Table S4. Total amount of transporters in rat organs calculated based on abundance data obtained from LC-MS/MS quantitative proteomics, western blot and ELISA techniques**

| Organ | Bcrp (μg)[a] | Mrp2 (μg)[d] | Mrp3 (μg)[f] | Oatp1b2 (μg)[i] |
|---|---|---|---|---|
| Duodenum | 0.66[b] | 0.03 | 0.20 | NA |
| Jejunum | 10.70 | 0.74 | 4.37 | NA |
| Total Ileum | 0.93 | 0.20 | 2.80 | NA |
| Colon | 0.43[b] | 0.03 | 0.45[g] | NA |
| Liver | 2.86[c] | 134.00[e] | 7.56[h] | 69.3 |

NA - Not Applicable; [a]Sharma et al., 2023 [11]; [b]calculated based on MacLean et al., 2008 [12]; [c]Li et al., 2009 [13]; [d]Gavins et al., 2023 [14]; [e]Li et al., 2009 [15]; [f]calculated with respect to LC-MS/MS based quantitative proteomic data from colon and western blot data from Rost et al., 2002 [16]; [g]Harbourt et al., 2009 [17]; [h]Fallon et al., 2016 [18]; Tu et al., 2021 [19]

**Table S5. Predicted rat tissue partition coefficient values**

| Organ | R&R predicted $K_p$ for GT | R&R predicted $K_p$ for GT-glu |
|---|---|---|
| $K_p$ Lung | 1.73 | 0.26 |
| $K_p$ Kidney | 1.29 | 0.18 |
| $K_p$ Liver | 1.36 | 0.13 |
| $K_p$ GIT | 2.62 | 0.20 |
| $K_p$ Rapidly perfused tissues | 9.64 | 0.80 |
| $K_p$ Slowly perfused tissues | 54.20 | 0.51 |

**ODE's**

### a. Arterial blood

$$\frac{dC_{arterial}}{dt} = \frac{1}{V_{arterial}}\left(Q_{lung} \times \frac{C_{lung} \times R_{B:P}}{K_{p,lung}} - Q_{tissue} \times C_{arterial}\right) \qquad \text{- Eq. S1}$$

Tissue = Kidney, liver, stomach, rapidly perfused tissues, slowly perfused tissues and gastrointestinal tract

### b. Venous blood

$$\frac{dC_{venous}}{dt} =$$

$$\frac{1}{V_{venous}}\left(Q_{tissue} \times \frac{C_{tissue} \times R_{B:P}}{K_{p,tissue}} + Q_{slowly\ perfused\ tissues} \times C_{slowly\ perfused\ tissues\ vascular} + Q_{liver} \times C_{liver\ vascular} - \right.$$

$$\left. Q_{lung} \times C_{venous}\right) \qquad \text{- Eq. S2}$$

Tissue = Kidney and rapidly perfused tissues

### c. Lung

$$\frac{dC_{lung}}{dt} = \frac{1}{V_{lung}}\left(Q_{lung} \times \left(C_{venous} - \frac{C_{lung} \times R_{B:P}}{K_{p,lung}}\right)\right) \qquad \text{- Eq. S3}$$

### d. Intestine

$$\frac{dC_{Int}}{dt} = \frac{1}{V_{Int}}\left(Q_{Int} \times \left(C_{arterial} - \frac{C_{Int} \times R_{B:P}}{K_{p,intestine}}\right) + \sum\left(k_{a,sys} \times A_{u,enterocyte}\right)\right) \qquad \text{- Eq. S4}$$

**e. Intestine_Glu**

$$\frac{dC_{Int\_Glu}}{dt} = \frac{1}{V_{Int}}\left(Q_{Int} \times \left(C_{arterial\_Glu} - \frac{C_{Int\_Glu} \times R_{B:P\_Glu}}{(K_{p,intestine\_Glu} \times K_{p,scalar\_Glu})}\right) + \sum\left(\frac{(J_{max,Mrp3} \times Mrp3 \times ISEF_{Mrp3}) \times C_{u,enterocyte\_Glu}}{K_{m,Mrp3} + C_{u,enterocyte\_Glu}}\right)\right)$$ - Eq. S5

**f. Kidney**

$$\frac{dC_{kidney}}{dt} = \frac{1}{V_{kidney}}\left(Q_{kidney} \times \left(C_{arterial} - \frac{C_{kidney} \times R_{B:P}}{K_{p,kidney}}\right)\right) - \left(\frac{GFR}{V_{kidney}}\right) \times F_{renal} \times C_{u,kidney}$$ - Eq. S6

**g. Kidney_Glu**

$$\frac{dC_{kidney\_Glu}}{dt} = \frac{1}{V_{kidney}}\left(Q_{kidney} \times \left(C_{arterial\_Glu} - \frac{C_{kidney\_Glu} \times R_{B:P}}{(K_{p,kidney\_Glu} \times K_{p,scalar\_Glu})}\right)\right) - \left(\frac{GFR}{V_{kidney}}\right) \times F_{renal\_Glu} \times C_{u,kidney\_Glu}$$ - Eq. S7

**h. Rapidly perfused tissues**

$$\frac{dC_{\text{rapidly perfused tissues}}}{dt} = \frac{1}{V_{\text{rapidly perfused tissues vascular}}}\left(Q_{\text{rapidly perfused tissues}} \times \left(C_{\text{arterial}} - \frac{C_{\text{rapidly perfused tissues}} \times R_{B:P}}{K_{p,\text{rapidly perfused tissues}} \times K_{p,\text{scalar}}}\right)\right)$$

- Eq. S8

## i. Slowly perfused tissues

$$\frac{dC_{\text{slowly perfused tissues vascular}}}{dt} = \frac{1}{V_{\text{slowly perfused tissues vascular}}}\left(\left(Q_{\text{slowly perfused tissues}} \times \left(C_{\text{arterial}} - C_{\text{slowly perfused tissues vascular}}\right)\right) - PS_{Sp} \times \left(\frac{C_{\text{slowly perfused tissues vascular}} \times f_{u,p}}{R_{B:P}} - \frac{C_{\text{slowly perfused tissues extravascular}} \times f_{u,p}}{(K_{p,\text{slowly perfused tissues}} \times K_{p,\text{scalar}})}\right)\right)$$

- Eq. S9

$$\frac{dC_{\text{slowly perfused tissues extravascular}}}{dt} = \frac{1}{V_{\text{slowly perfused tissues extravascular}}}\left(PS_{Sp} \times \left(\frac{C_{\text{slowly perfused tissues vascular}} \times f_{u,p}}{R_{B:P}} - \frac{C_{\text{slowly perfused tissues extravascular}} \times f_{u,p}}{K_{p,\text{slowly perfused tissues}} \times K_{p,\text{scalar}}}\right)\right)$$

- Eq. S10

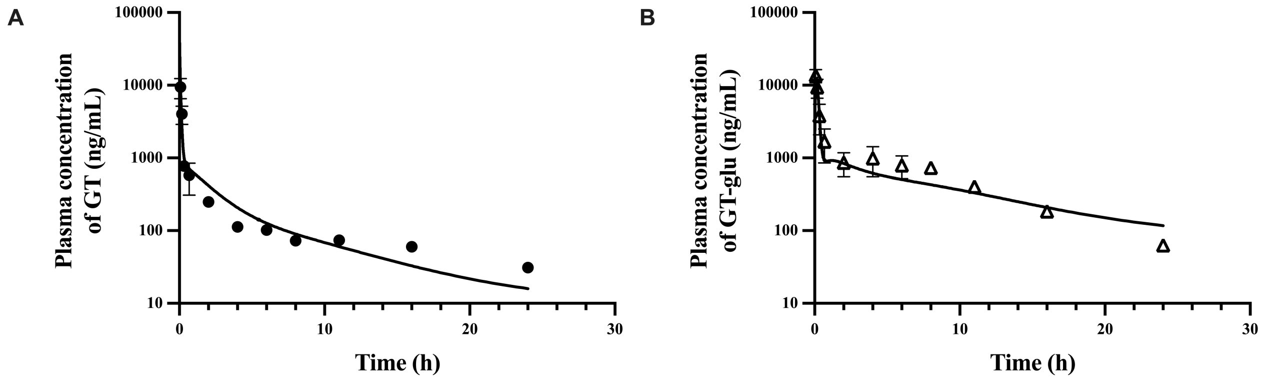


**Figure S1.** Plasma concentration of genistein (A) and its glucuronide (B) representing model fitting after intravenous administration of 12.5 mg/kg genistein. Solid lines are predicted profiles. Closed circles and open triangles are observed data for genistein (A) and genistein glucuronide (B), respectively.

A

C

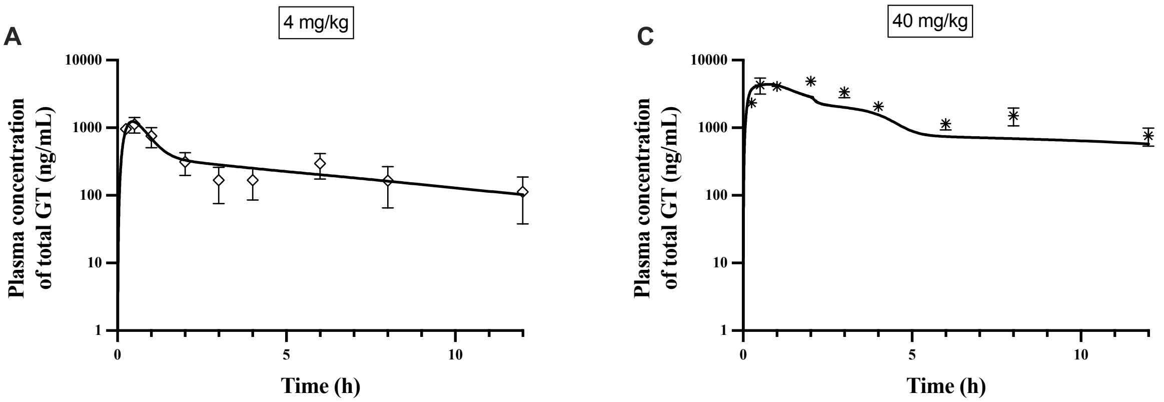


B

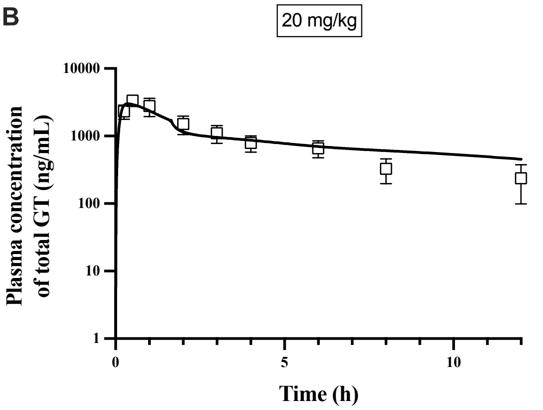


D

| | Predicted | Observed | FE[a] |
|---|---|---|---|
| ***4 mg/kg*** | | | |
| $C_{max}$ (ng/mL) | 1238 | 1126 | 1.1 |
| $AUC_{0\ to\ last}$ (ng.h/mL) | 3998 | 3395 | 1.2 |
| $t_{1/2}$ (hour) | 6 | 5 | 1.3 |
| ***20 mg/kg*** | | | |
| $C_{max}$ (ng/mL) | 3003 | 3339 | 0.9 |
| $AUC_{0\ to\ last}$ (ng.h/mL) | 14411 | 10791 | 1.3 |
| $t_{1/2}$ (hour) | 8 | 4 | 1.8 |
| ***40 mg/kg*** | | | |
| $C_{max}$ (ng/mL) | 4393 | 4878 | 0.9 |
| $AUC_{0\ to\ last}$ (ng.h/mL) | 21575 | 25288 | 0.9 |
| $t_{1/2}$ (hour) | 8 | 5 | 1.5 |

**Figure S2.** Plasma concentration of total genistein concentration after oral administration of 4 mg/kg (A), 20 mg/kg (B) and 40 mg/kg (C) genistein. (D) [a]FE = Fold error (predicted value/observed value) $0.5 \leq FE \leq 2.0$. Solid lines are predicted profiles. Open diamonds, open squares and asterisk are observed total genistein concentrations for 4, 20 and 40 mg/kg, respectively.

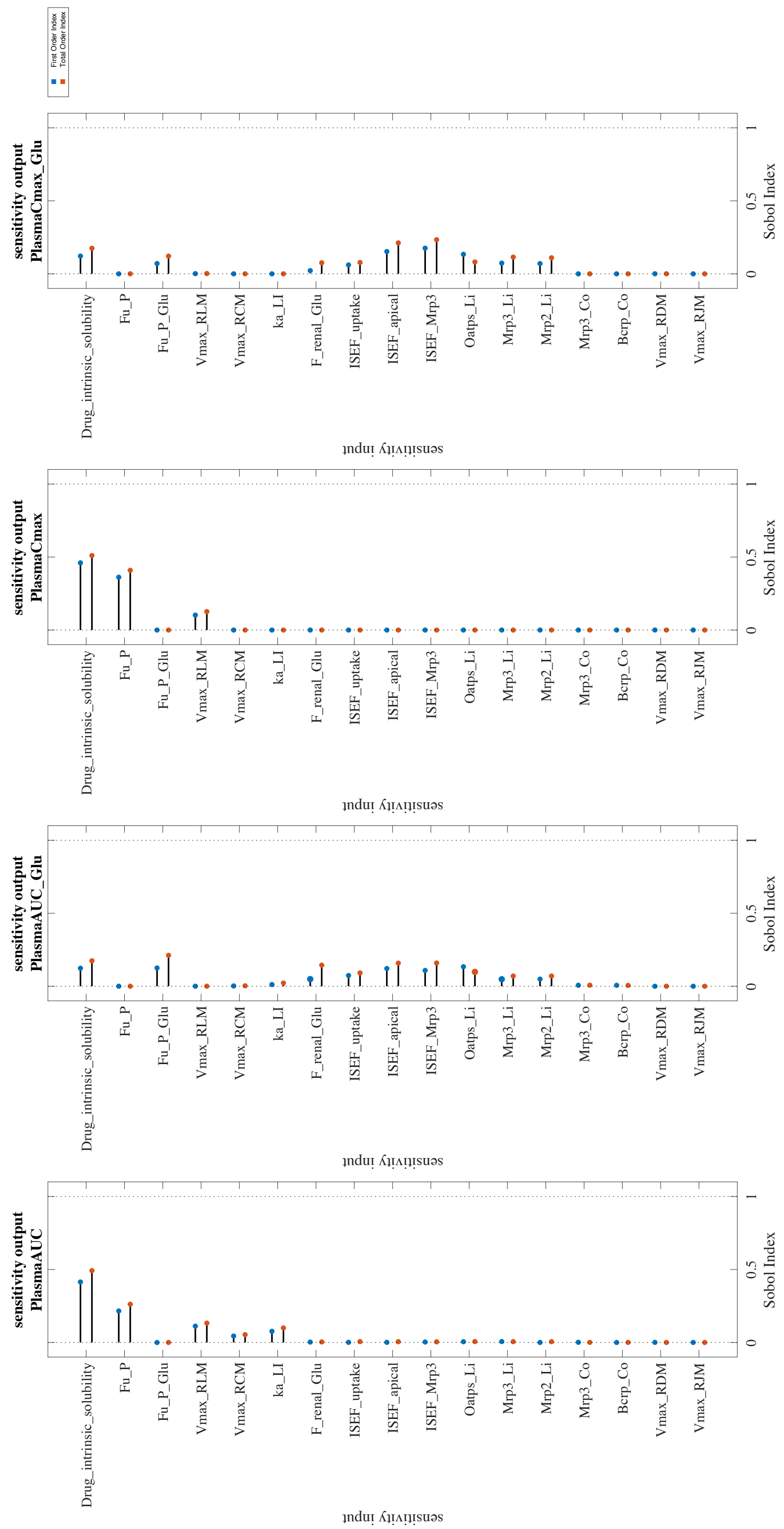


**Figure S3.** GSA Sobol indices showing sensitivities of GT and GT-glu plasma AUC and $C_{max}$ towards model parameters. Lines with blue dots and orange dots are first order and total order Sobol indices, respectively.

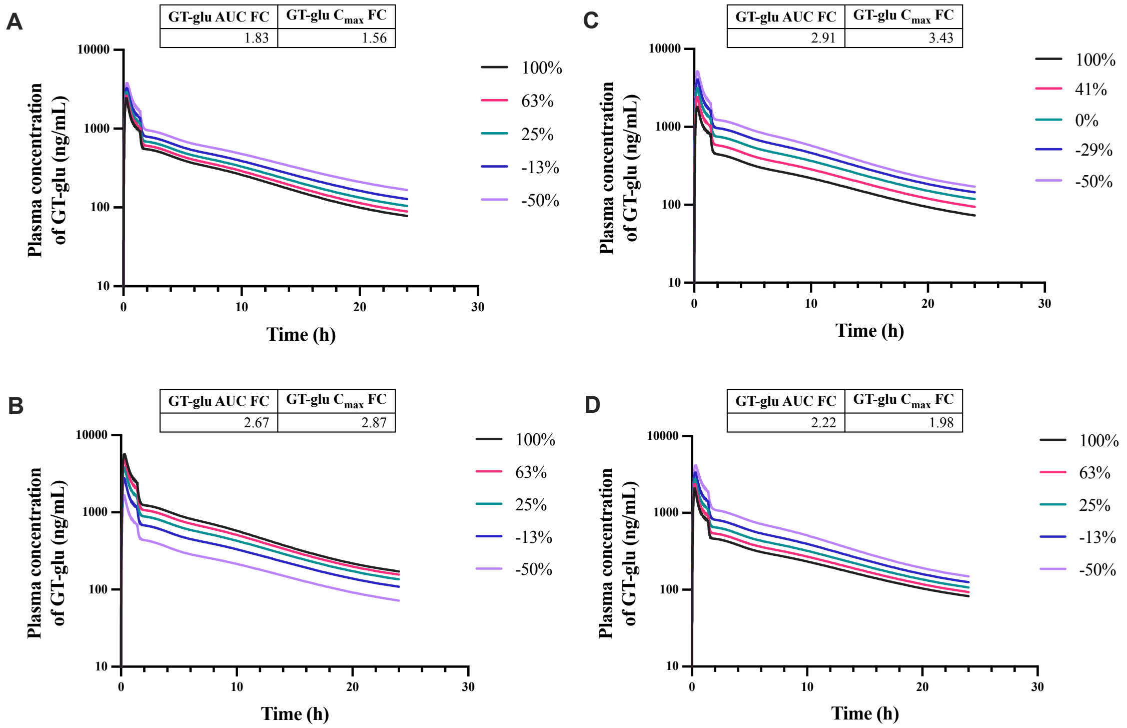


**Figure S4.** Sensitivity of genistein glucuronide plasma concentration towards (A) renal excretion of glucuronide, (B) ISEF_apical, (C) ISEF_Mrp3 and (D) ISEF_uptake. Fold change (FC) = variation in PK parameter value within 2-fold range.

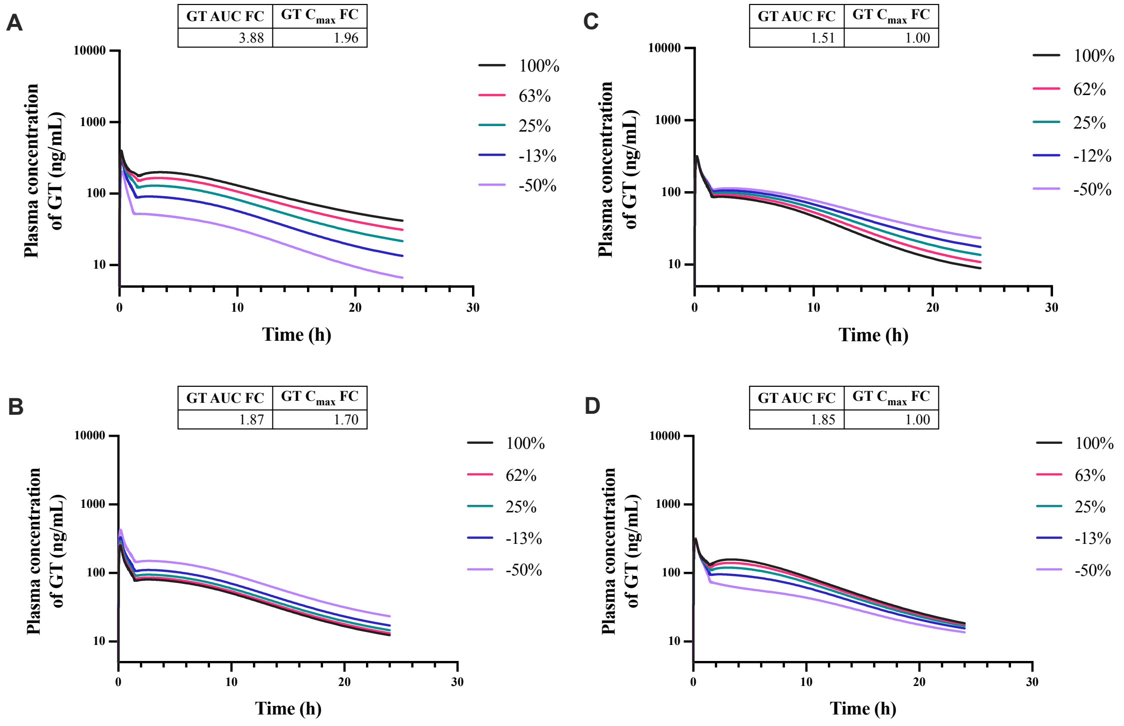


**Figure S5.** Sensitivity of genistein plasma concentration towards (A) solubility of genistein, (B) liver metabolism, (C) colon metabolism and (D) absorption of regenerated genistein in caecum. Fold change (FC) = variation in PK parameter value within 2-fold range.

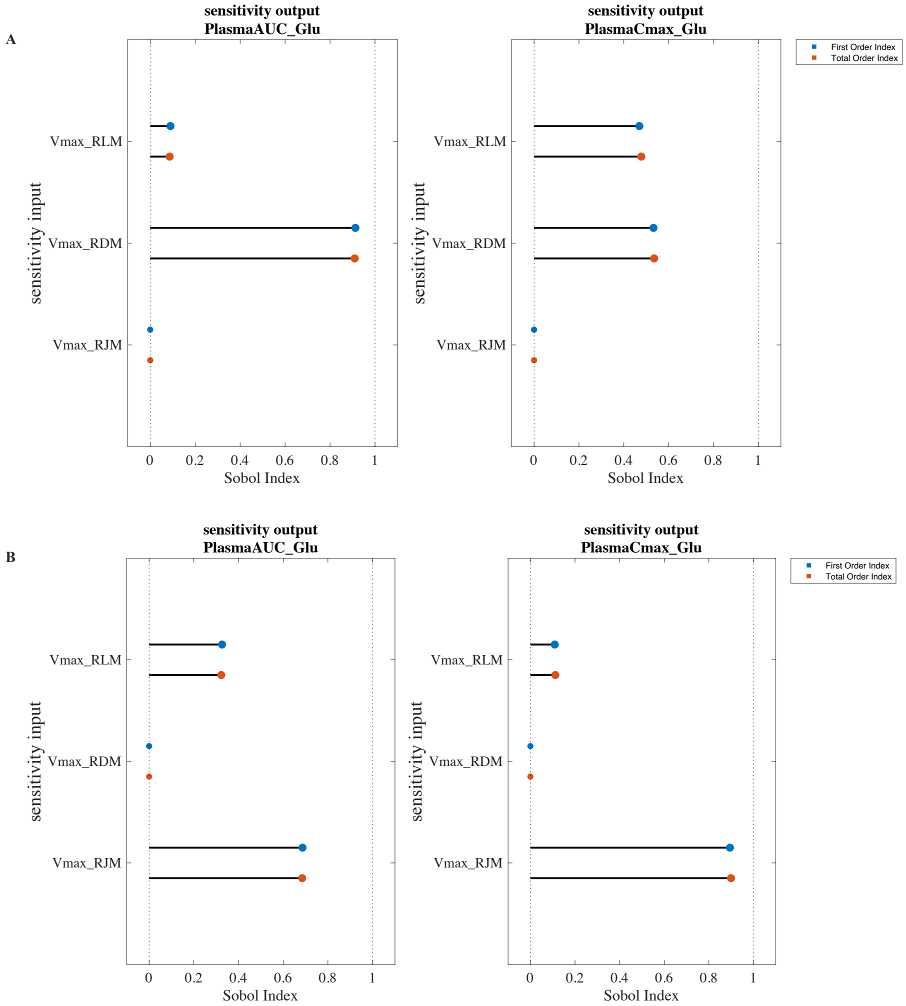


**Figure S6.** GSA Sobol indices showing sensitivity of plasma AUC and $C_{max}$ of genistein glucuronide (GT-glu) towards liver and intestinal metabolism after oral administration of 12.5 mg/kg genistein. A) PO dosing, B) Jejunal dosing, and lines with blue dots and orange dots are first order and total order Sobol indices, respectively.

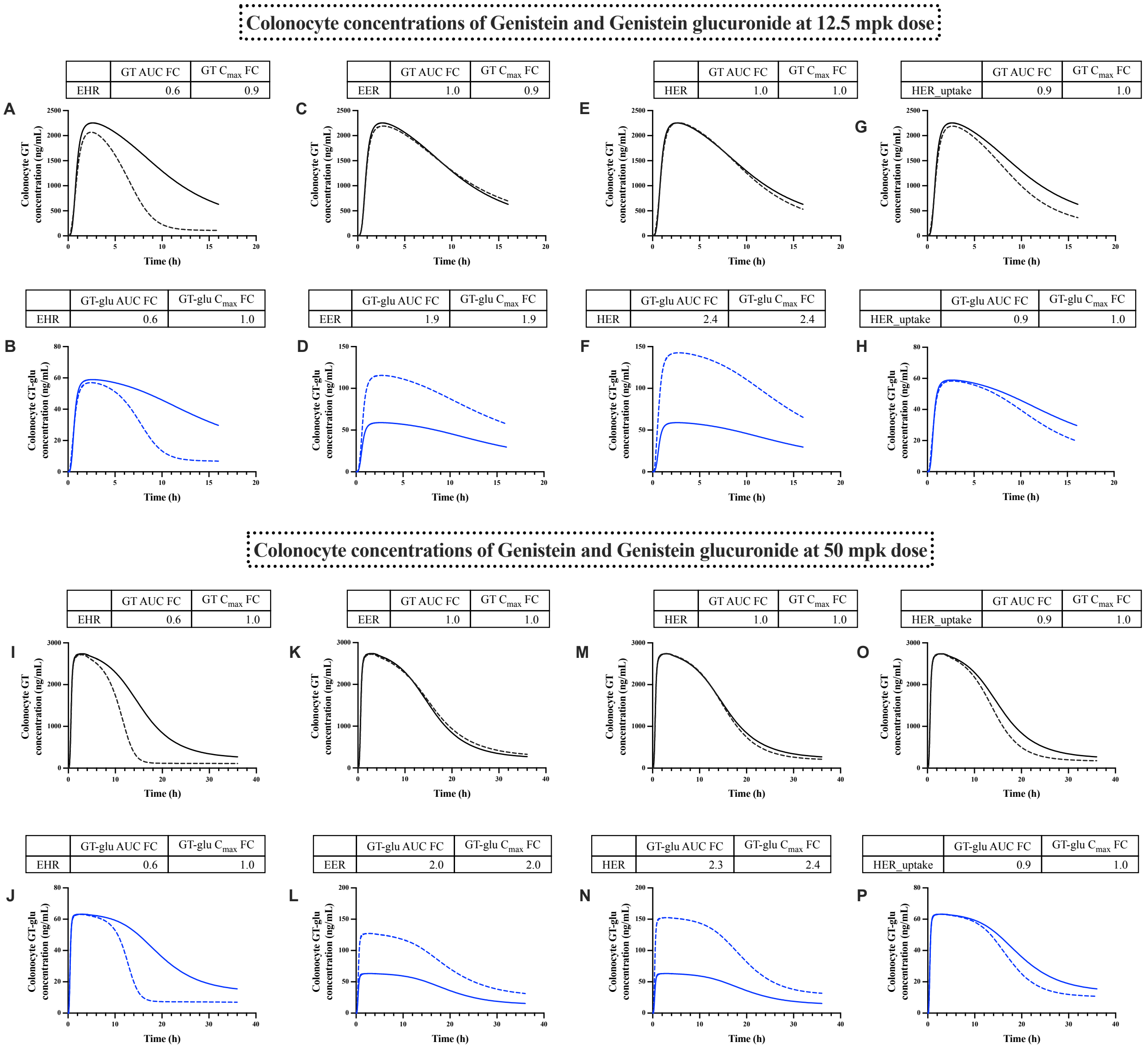


**Figure S7.** Influence of recycling mechanisms on colonocyte concentrations of genistein and genistein glucuronide after oral administration of 12.5 mg/kg (A to H) and 50 mg/kg (I to P) of genistein. The graphs A, B and I, J represent the effect of EHR on colonocyte concentrations of genistein and its glucuronide at 12.5 mg/kg and 50 mg/kg doses, respectively. The graphs C, D and K, L represent the effect of EER on colonocyte concentrations of genistein and its glucuronide at 12.5 mg/kg and 50 mg/kg doses, respectively. The graphs E, F and M, N represent the effect of HER on colonocyte concentrations of genistein and its glucuronide at 12.5 mg/kg and 50 mg/kg doses, respectively. The graphs E, F and O, P represent the effect of HER_uptake on colonocyte concentrations of genistein and its glucuronide at 12.5 mg/kg and 50 mg/kg doses, respectively. Solid lines are predicted profiles and dashed lines are predictions without the listed mechanisms. Black and blue colors represent genistein and genistein glucuronide, respectively. Fold change (FC) = without recycling mechanism/with recycling mechanism.

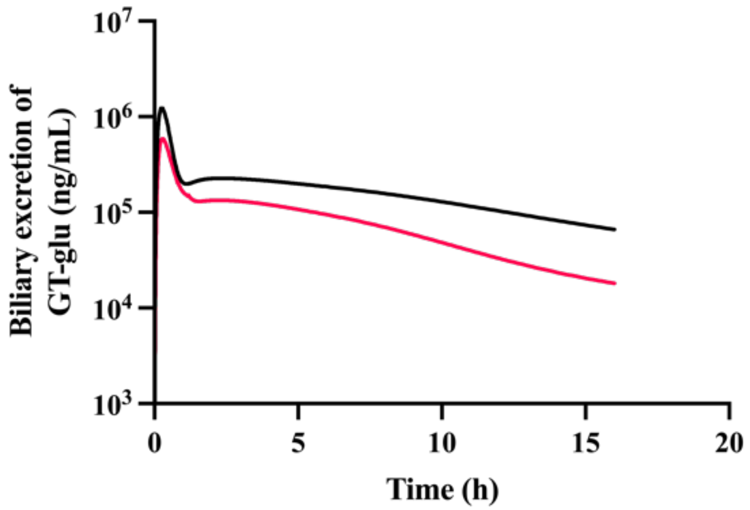


**Figure S8.** Biliary profiles of genistein glucuronide with 10-fold decreased apical efflux transporter activity (red lines) and original values (black lines) activity.

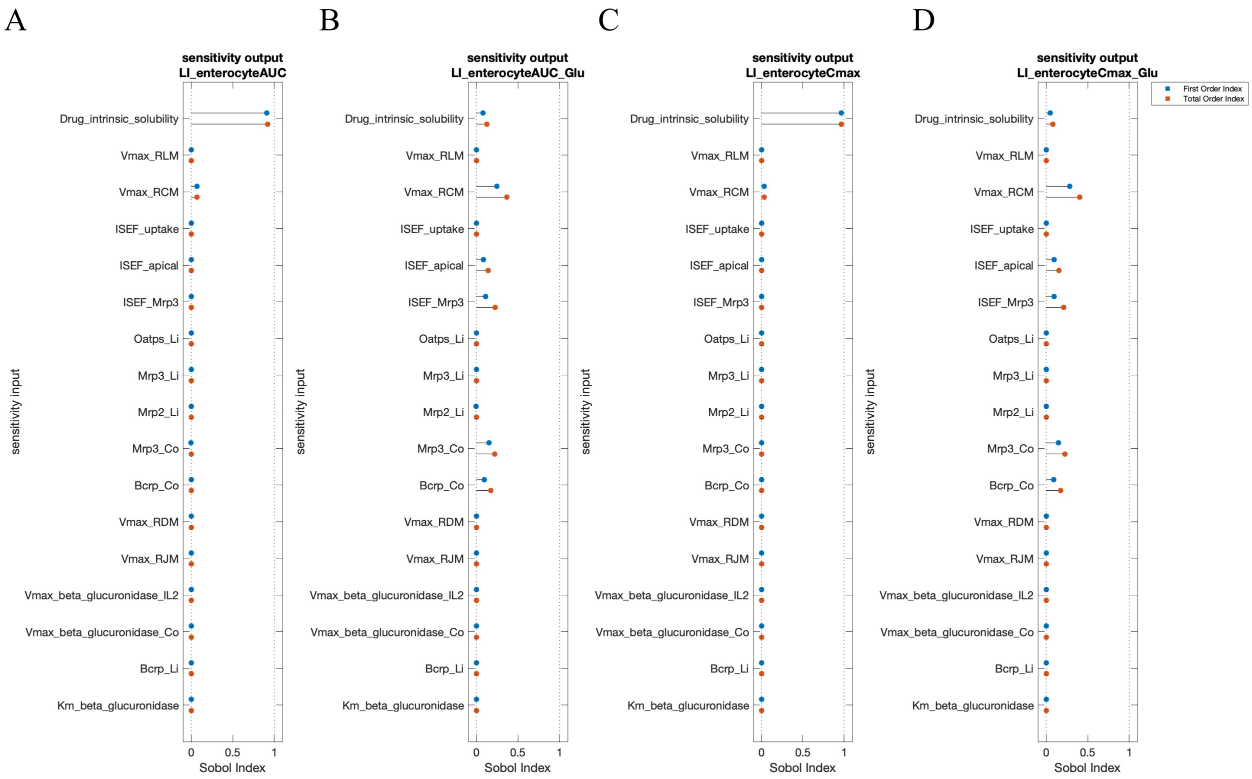


**Figure S9.** GSA Sobol indices showing sensitivity of colonocyte GT (A and C) and GT-glu (B and D) AUC (A and B) and $C_{max}$ (C and D) towards various model parameters after oral administration of genistein. Lines with blue dots and orange dots are first order and total order Sobol indices, respectively.

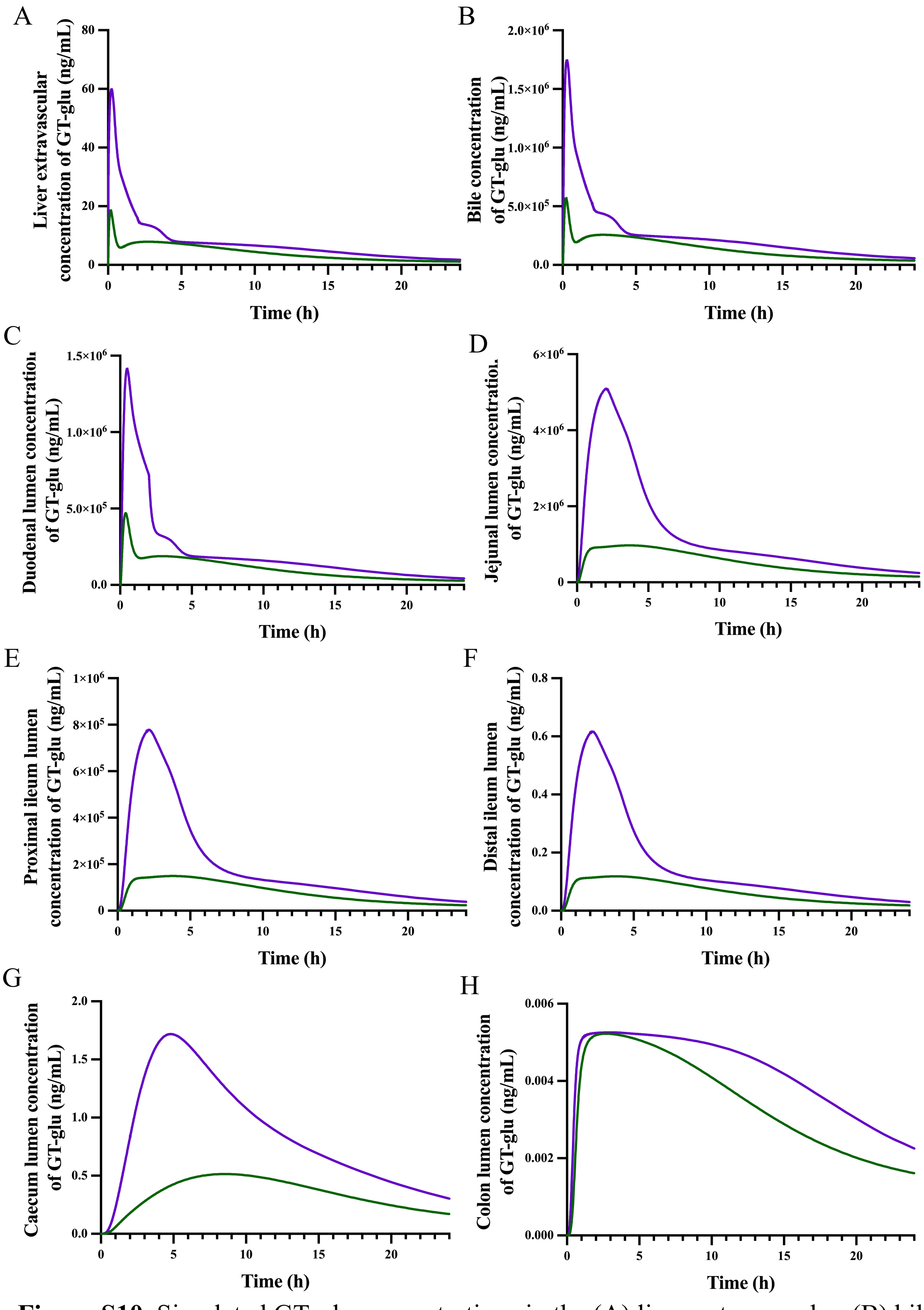


**Figure S10.** Simulated GT-glu concentrations in the (A) liver extravascular, (B) bile, and lumens of (C) duodenum, (D) jejunum, (E) proximal ileum, (F) distal ileum, (G) caecum, and (H) colon after oral administration of 12.5 mg/kg (green lines) or 50 mg/kg (violet lines) of genistein.

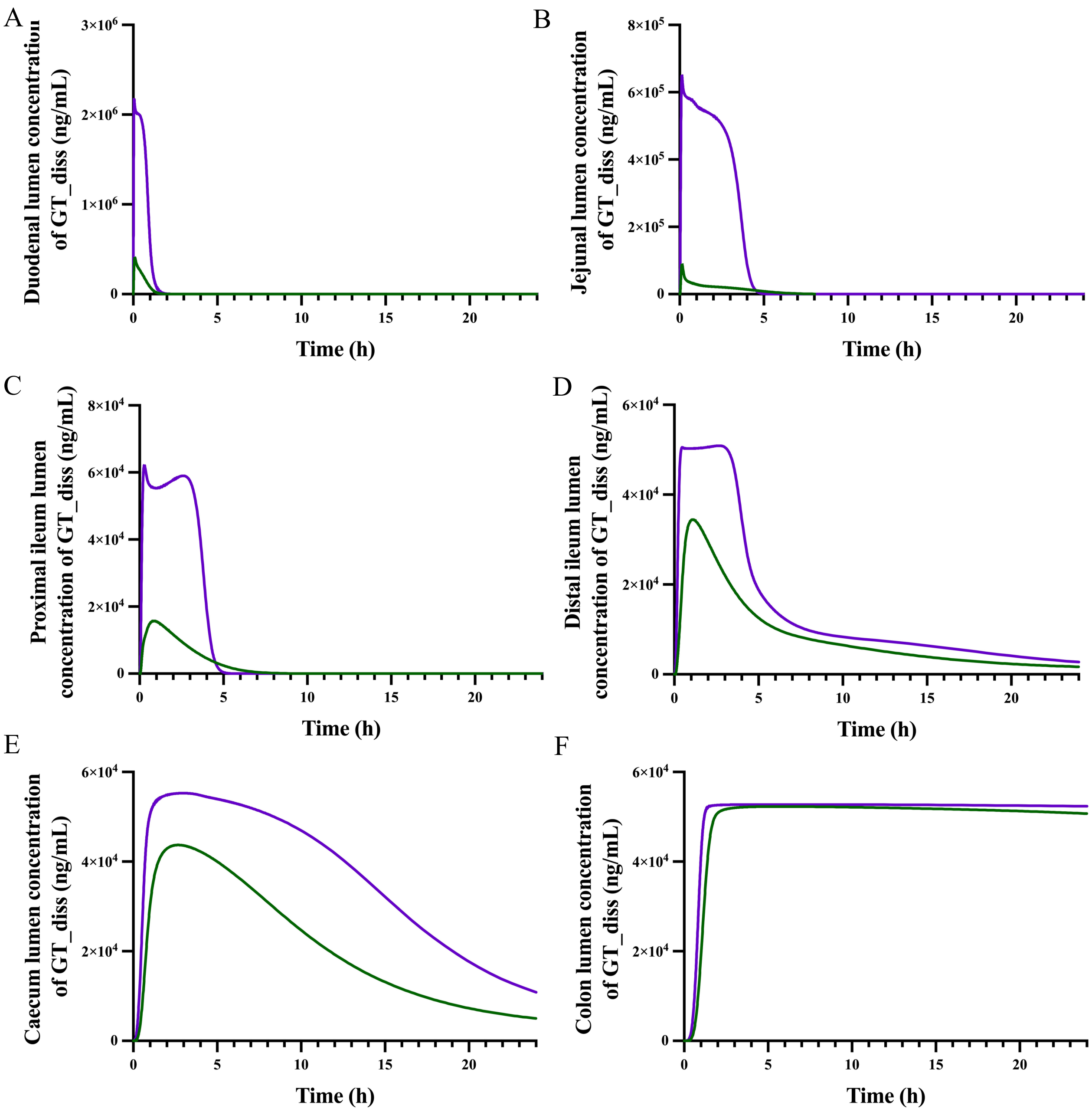


**Figure S11.** Simulated dissolved GT concentrations in the lumens of (A) duodenum, (B) jejunum, (C) proximal ileum, (D) distal ileum, (E) caecum, and (F) colon after oral administration of 12.5 mg/kg (green lines) or 50 mg/kg (violet lines) of genistein.

## References


1. Brown, R.P.; Delp, M.D.; Lindstedt, S.L.; Rhomberg, L.R.; Beliles, R.P. Physiological parameter values for physiologically based pharmacokinetic models. *Toxicology and industrial health* **1997**, *13*, 407-484.
2. Davies, B.; Morris, T. Physiological parameters in laboratory animals and humans. *Pharm Res* **1993**, *10*, 1093-1095, doi:10.1023/a:1018943613122.
3. McConnell, E.L.; Basit, A.W.; Murdan, S. Measurements of rat and mouse gastrointestinal pH, fluid and lymphoid tissue, and implications for in-vivo experiments. *J Pharm Pharmacol* **2008**, *60*, 63-70, doi:10.1211/jpp.60.1.0008.
4. Blouin, A.; Bolender, R.P.; Weibel, E.R. Distribution of organelles and membranes between hepatocytes and nonhepatocytes in the rat liver parenchyma. A stereological study. *The Journal of cell biology* **1977**, *72*, 441-455.
5. Musther, H.; Harwood, M.D.; Yang, J.; Turner, D.B.; Rostami-Hodjegan, A.; Jamei, M. The constraints, construction, and verification of a strain-specific physiologically based pharmacokinetic rat model. *Journal of pharmaceutical sciences* **2017**, *106*, 2826-2838.
6. Peters, S.A. Evaluation of a generic physiologically based pharmacokinetic model for lineshape analysis. *Clinical pharmacokinetics* **2008**, *47*, 261-275.
7. DeSesso, J.; Jacobson, C. Anatomical and physiological parameters affecting gastrointestinal absorption in humans and rats. *Food and chemical toxicology* **2001**, *39*, 209-228.
8. Kirman, C.; Hays, S.; Aylward, L.; Suh, M.; Harris, M.; Thompson, C.; Haws, L.; Proctor, D. Physiologically based pharmacokinetic model for rats and mice orally exposed to chromium. *Chemico-biological interactions* **2012**, *200*, 45-64.
9. Chen, J.; Wang, S.; Jia, X.; Bajimaya, S.; Lin, H.; Tam, V.H.; Hu, M. Disposition of flavonoids via recycling: comparison of intestinal versus hepatic disposition. *Drug metabolism and Disposition* **2005**, *33*, 1777-1784.
10. Ebuzoeme, C.; Etim, I.; Ikimi, A.; Song, J.; Du, T.; Hu, M.; Liang, D.; Gao, S. Glucuronides hydrolysis by intestinal microbial β-glucuronidases (GUS) is affected by sampling, enzyme preparation, buffer pH, and species. *Pharmaceutics* **2021**, *13*, 1043.
11. Sharma, S.; Singh, D.K.; Mettu, V.S.; Yue, G.; Ahire, D.; Basit, A.; Heyward, S.; Prasad, B. Quantitative characterization of clinically relevant drug-metabolizing enzymes and transporters in rat liver and intestinal segments for applications in PBPK modeling. *Molecular Pharmaceutics* **2023**, *20*, 1737-1749.
12. MacLean, C.; Moenning, U.; Reichel, A.; Fricker, G. Closing the gaps: a full scan of the intestinal expression of p-glycoprotein, breast cancer resistance protein, and multidrug resistance-associated protein 2 in male and female rats. *Drug Metabolism and Disposition* **2008**, *36*, 1249-1254.
13. Li, N.; Palandra, J.; Nemirovskiy, O.V.; Lai, Y. LC− MS/MS mediated absolute quantification and comparison of bile salt export pump and breast cancer resistance protein in livers and hepatocytes across species. *Analytical chemistry* **2009**, *81*, 2251-2259.
14. Gavins, F.K.; Dou, L.; Qin, Y.; Madla, C.M.; Murdan, S.; Basit, A.W.; Mai, Y.; Orlu, M. Prandial state and biological sex modulate clinically relevant efflux transporters to different extents in Wistar and Sprague Dawley rats. *Biomedicine & Pharmacotherapy* **2023**, *160*, 114329.

15. Li, N.; Zhang, Y.; Hua, F.; Lai, Y. Absolute difference of hepatobiliary transporter multidrug resistance-associated protein (MRP2/Mrp2) in liver tissues and isolated hepatocytes from rat, dog, monkey, and human. *Drug metabolism and disposition* **2009**, *37*, 66-73.
16. Rost, D.; Mahner, S.; Sugiyama, Y.; Stremmel, W. Expression and localization of the multidrug resistance-associated protein 3 in rat small and large intestine. *American journal of physiology-gastrointestinal and liver physiology* **2002**, *282*, G720-G726.
17. Harbourt, D.E. An investigation into the role of glucuronidation on the disposition and toxicity of mycophenolic acid using targeted quantitative proteomics. The University of North Carolina at Chapel Hill, 2009.
18. Fallon, J.K.; Smith, P.C.; Xia, C.Q.; Kim, M.-S. Quantification of four efflux drug transporters in liver and kidney across species using targeted quantitative proteomics by isotope dilution NanoLC-MS/MS. *Pharm Res-Dordr* **2016**, *33*, 2280-2288.
19. Tu, Y.; Wang, L.; Rong, Y.; Tam, V.; Yin, T.; Gao, S.; Singh, R.; Hu, M. Hepatoenteric recycling is a new disposition mechanism for orally administered phenolic drugs and phytochemicals in rats. *Elife* **2021**, *10*, e58820.
